# Trends in Antidiabetic Drug Discovery: FDA Approved Drugs, New Drugs in Clinical Trials and Global Sales

**DOI:** 10.3389/fphar.2021.807548

**Published:** 2022-01-19

**Authors:** Amelia D. Dahlén, Giovanna Dashi, Ivan Maslov, Misty M. Attwood, Jörgen Jonsson, Vladimir Trukhan, Helgi B. Schiöth

**Affiliations:** ^1^ Functional Pharmacology, Department of Neuroscience, Uppsala University, Uppsala, Sweden; ^2^ Department of Biology, Lomonosov Moscow State University, Moscow, Russia; ^3^ Russia Institute of Translational Medicine and Biotechnology, I. M. Sechenov First Moscow State Medical University, Moscow, Russia

**Keywords:** diabetes mellitus, FDA approved, antihyperglycemics, clinical developments, efficacy and safety, global trends

## Abstract

Type 2 diabetes mellitus (T2DM) continues to be a substantial medical problem due to its increasing global prevalence and because chronic hyperglycemic states are closely linked with obesity, liver disease and several cardiovascular diseases. Since the early discovery of insulin, numerous antihyperglycemic drug therapies to treat diabetes have been approved, and also discontinued, by the United States Food and Drug Administration (FDA). To provide an up-to-date account of the current trends of antidiabetic pharmaceuticals, this review offers a comprehensive analysis of the main classes of antihyperglycemic compounds and their mechanisms: insulin types, biguanides, sulfonylureas, meglitinides (glinides), alpha-glucosidase inhibitors (AGIs), thiazolidinediones (TZD), incretin-dependent therapies, sodium-glucose cotransporter type 2 (SGLT2) inhibitors and combinations thereof. The number of therapeutic alternatives to treat T2DM are increasing and now there are nearly 60 drugs approved by the FDA. Beyond this there are nearly 100 additional antidiabetic agents being evaluated in clinical trials. In addition to the standard treatments of insulin therapy and metformin, there are new drug combinations, e.g., containing metformin, SGLT2 inhibitors and dipeptidyl peptidase-4 (DPP4) inhibitors, that have gained substantial use during the last decade. Furthermore, there are several interesting alternatives, such as lobeglitazone, efpeglenatide and tirzepatide, in ongoing clinical trials. Modern drugs, such as glucagon-like peptide-1 (GLP-1) receptor agonists, DPP4 inhibitors and SGLT2 inhibitors have gained popularity on the pharmaceutical market, while less expensive over the counter alternatives are increasing in developing economies. The large heterogeneity of T2DM is also creating a push towards more personalized and accessible treatments. We describe several interesting alternatives in ongoing clinical trials, which may help to achieve this in the near future.

## 1 Introduction

This year marks the 100th anniversary of the pioneering experiments by Banting, and later on Best, Macleod and Collip, that led to the discovery of insulin to treat diabetes (Quianzon and Cheikh, 2012). Since then, the life expectancy and clinical prognosis for diabetic patients have drastically improved. However, the prevalence of the progressive metabolic disease, caused by genetic and environmental factors, continues to grow at alarming rates (Kharroubi and Darwish, 2015). By 2045, 784 million adults are estimated to be diagnosed with type 1 or type 2 diabetes mellitus (International Diabetes Federation, 2021).

Type 1 diabetes mellitus is an autoimmune disease. It is characterized by a loss of pancreatic β-cells, which impairs insulin production (Ndisang et al., 2017). In contrast, obesity and sedentary ways of life are major risk factors for developing type 2 diabetes mellitus (T2DM), where high glucose concentrations in the blood (hyperglycemia) are caused by insensitivity and defective pancreatic β-cells (Halban et al., 2014; Ndisang et al., 2017). T2DM is the most common form of diabetes and accounts for ∼90% of all diabetic cases (Kharroubi and Darwish, 2015). This is largely attributed to the drastic lifestyle changes which have taken place in the past 30 years, whereby technological advances have made processed and calorically dense foods readily available and made it easier to be productive while sedentary. Hyperglycemia causes micro and macro-vascular complications, which are often the cause of mortality among the patients. Microvascular complications affect small arteries and vessels and result in neuropathy, nephropathy, and retinopathy, whereas macrovascular complications involve large arteries and vessels and result in cardiovascular diseases, stroke, and peripheral artery disease (Chawla et al., 2016). As the symptoms develop gradually, the disease has often reached mid-stages before a diagnosis has been made and any drug treatment initiated (Kharroubi and Darwish, 2015). There is currently no cure for the disease, but modern diagnostic algorithms are allowing earlier detection of T2DM, which is essential for prescribing patient-appropriate antidiabetic treatment.

The complexity of T2DM has prompted a significant interest in developing new pharmaceutical therapies to control diabetes. A broad range of drug therapies has been approved by the United States Food and Drug Administration (FDA) that address different biological systems and mechanisms implicated in the disease (Gourgari et al., 2017). In this rapidly developing area of pharmacology, hundreds of compounds with antihyperglycemic activity have been discovered in nature or synthesized in recent years, many of which are currently undergoing clinical trials (Artasensi et al., 2020). Furthermore, there are several agents in different stages of clinical development that target both established pathways and novel mechanisms of actions for antidiabetic drugs. Excellent reviews have been published that focus on specific aspects of diabetes drug discovery, for example on FDA-approved antihyperglycemic agents (Gourgari et al., 2017; Artasensi et al., 2020), specific delivery systems to improve drug efficacy (Veiseh et al., 2015), and on targeting particular pathways that are being investigated in clinical development. However, the landscape of approved agents for the treatment of diabetes is dynamic, with both drug approvals and discontinuations taking place (Scheen et al., 2017; Chikara et al., 2018). Hence, an updated comprehensive analysis is needed to examine the current state of diabetes drug discovery and search for new diabetology approaches.

This analysis aims to shed light on the current trends of FDA-approved antidiabetic pharmaceuticals and all the drugs to treat diabetes in clinical development from 2015 to 2020. We will also discuss the mechanisms of action of the main classes of antihyperglycemic compounds and present new and promising, but not yet approved, therapeutic approaches for treating T2DM.

## 2 Analysis of Antihyperglycemic Agents

We have compiled a comprehensive dataset that includes the unique antihypertensive agents that the FDA have approved as monotherapies and combination therapies. We have also collated and manually curated the clinical agents registered in ClinicalTrials.gov from 2015 through 2020 for diabetes treatment. The FDA-approved antidiabetic drugs were verified using Drugs@FDA.gov. This official United States government database lists most prescription and over-the-counter drug products approved by the FDA and is updated daily. Information on the agents in clinical trials was obtained from our previously published datasets of drug-target interactions (Rask-Andersen et al., 2014; Hauser et al., 2017; Attwood et al., 2018; Attwood et al., 2020; Atwood et al., 2021), publicly available resources including CenterWatch Weekly (www.centerwatch.com), and literature reviews. Additionally, keyword searches were performed in ClinicalTrials.gov using the term “diabetes” for condition and inclusive of the last 5 years. ClinicalTrials.gov is the United States repository for registered clinical trials and contains more than 360,000 research studies (clinicaltrials.gov). Our analysis focuses on novel agents in clinical trials and excludes drug entries that are chemical duplicates of previously approved drugs. We manually curated the mechanism of action for each agent, the primary therapeutic targets and classified the molecular type. The dataset is provided to the public for further analysis by the scientific community in Supplementary Table S1.

**FIGURE 1:**
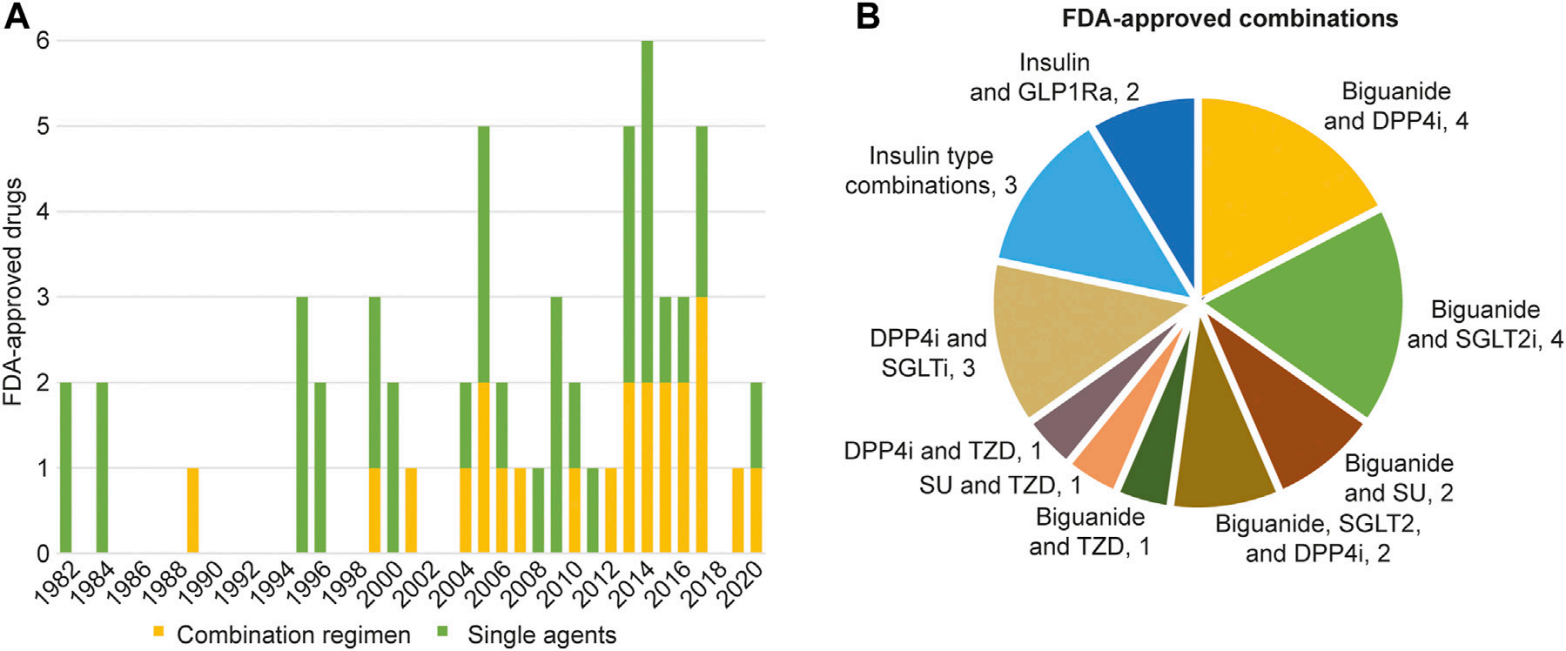
FDA-approved monotherapies and combination regimens. **(A)** The current 59 FDA-approved anti-diabetic agents. The data includes the year of first approval for new molecular entities and unique combinations. Single agents are indicated in green while combination regimens are in yellow. **(B)** FDA-approved unique combinations. Data compiled and verified using the United States Drugs@FDA resource. DPP4i, Dipeptidyl peptidase 4 (DPP4) inhibitor; GLP-1Ra, Glucagon-like peptide-1 (GLP-1) receptor agonist; SGLT2i, Sodium-glucose co-transporter-2 (SGLT2) inhibitor; SU, Sulfonylureas; TZD, Thiazolinediones.

The FDA has approved 59 unique antihyperglycemic drugs since human insulin (Humulin) approval in 1982 (Table 1). The approved drugs include 36 new molecular entities (NMEs) as monotherapies and 23 unique drug combinations of two or more antihyperglycemic agents (Figure 1A). Most recently approved NMEs that are monotherapies target already established molecular pathways that other authorized antihyperglycemic agents have validated. For example, the most recent new molecular target is sodium-glucose cotransporter type 2 (SGLT2), approved in 2014. In addition, however, the approvals of combination regimens that target multiple pathways for diabetes mellitus management have been increasing (Figure 1B).

**TABLE 1:** FDA-approved anti-diabetic agents.

Type	Agent(s)	S or C	Mechanism of action	FDA approval date
Insulin types	Insulin human (Humulin N)	s	Intermediate	1982
	Insulin human (Humulin R)	s	Short-acting	1982
	Insulin lispro (Humalog)	s	Rapid-acting	1996
	Insulin glargine (Lantus)	s	Long-acting	2000
	Insulin aspart (Novolog)	s	Rapid-acting	2000
	Insulin glulisine (Apidra)	s	Rapid-acting	2004
	Insulin detemir (Levemir)	s	Long-acting	2005
	Insulin degludec (Tresiba)	s	Ultra-long-acting	2015
	Insulin lispro-AABC (Lyumjev)	s	Rapid-acting	2020
	Humulin 70/30	c	Intermediate	1989
	Humalog mix 75/25, 50/50	c	Rapid; intermediate	1999
	Novolog 70/30	c	Rapid; intermediate	2001
Insulin and GLP-1R combination	Insulin degludec; Liraglutide (Xultophy)	c	Improve glycemic control, long-acting	2016
	Insulin glargine; Lixisenatide (Soliqua)	c		2016
SU	Glyburide (Glynase)	s	Stimulate insulin secretion	1984
	Glipizide (Glucotrol)	s		1984
	Glimepiride (Amaryl)	s		1995
Alpha-glucosidase inhibitors	Acarbose (Precose)	s	Prevent the digestion of carbohydrates, improve glycemic control	1995
	Miglitol (Glyset)	s		1996
TZD	Rosiglitazone (Avandia)	s	Insulin sensitizer	1999
	Pioglitazone (Actos)	s		1999
Biguanide	Metformin (Glumetza)	s	Inhibit gluconeogenesis, insulin sensitizer, pleotropic effects	1995
Biguanide and SU combination	Metformin; Glyburide	c	Insulin sensitizer, Decrease gluconeogenesis	2004
	Metformin; Glipizide	c		2005
Biguanide and TZD combination	Metformin; Pioglitazone (Actoplus Met)	c	Reduce insulin resistance	2005
Biguanide DPP-4i combination	Metformin; Sitagliptin (Janumet)	c	Prevent breakdown of GLP-1 and GIP, stimulate insulin secretion and decrease glucagon release from the pancreas	2007
	Metformin; Sitagliptin (Kombiglyze XR)	c		2010
	Metformin; Linagliptin (Jentadueto)	c		2012
	Metformin; Alogliptin (Kazano)	c		2013
Biguanide and SGLT2i combination	Metformin; Canagliglozin (Invokamet)	c	Reduces blood glucose by blocking glucose reabsorption in the kidney	2014
	Metformin; Dapagliflozin (Xigduo XR)	c		2014
	Metformin; Empagliflozin (Synjardy)	c		2015
	Metformin; Ertugliflozin (Segluromet)	c		2017
Biguanide, SGLT2i and DPP-4i combination	Metformin; Saxagliptin; Dapagliflozin (Qternmet XR)	c	Increases insulin production and decreases the rate of gluconeogenesis in the liver	2019
	Metformin; Linagliptin; Empagliflozin	c		2020
Amylin analogue	Pramlintide (Symlin)	s	Short-acting	2005
GLP1R agonist	Exenatide (Byetta)	s	Increase insulin secretion and inhibit glucagon secretion from pancreatic islet cells	2005
	Liraglutide (Victoza)	s		2010
	Dulaglutide (Trulicity)	s		2014
	Albiglutide (Tanzeum)	s		2014
	Lixisenatide (Adlyxin)	s		2016
	Semaglutide (Ozempic)	s		2017
SU and TZD combination	Glimepiride; Pioglitazone (Duetact)	c	Insulin secretagogues	2006
DPP-4i	Sitagliptin (Januvia)	s	Inhibit glucagon release and increase insulin secretion	2006
	Saxagliptin (Onglyza)	s		2009
	Linagliptin (Trajenta)	s		2011
	Alogliptin (Nesina)	s		2013
Meglitinides	Nateglinide (Starlix)	s	Stimulate insulin secretion	2009
	Repaglinide (Prandin)	s		2013
Bile acid sequestrant	Colesevelam	s	Clearance of LDL cholesterol	2008
Dopamine Receptor agonist	Bromocriptine (Cycloset)	s	Stimulate hypothalamic dopamine D2 receptors, improve glycemic control	2009
SGLT2i	Canagliflozin (Invokana)	s	Block glucose reabsorption in the kidney	2013
	Dapagliflozin (Farxiga)	s		2014
	Empagliflozin (Jardiance)	s		2014
	Ertugliflozin (Steglatro)	s		2017
DPP-4i and TZD	Alogliptin; Pioglitazone (Oseni)	c	Reduce insulin resistance and decrease gluconeogenesis	2013
DPP-4i and SGLT2i	Linagliptin; Empagliflozin (Glyxambi)	c	Block glucose reabsorption, inhibit glucagon release and increase insulin secretion	2015
	Sitagliptin; Ertugliflozin (Steglujan)	c		2017
	Saxagliptin; Dapagliflozin (Qtern)	c		2017

C, combination therapy; DPP-4i, Dipeptidyl peptidase-4 (DPP-4) inhibitor; GIP, gastric inhibitory peptide; GLP-1Ra, Glucagon-like peptide-1 (GLP-1) receptor agonist; S, single therapy; SGLT2i, Sodium-glucose co-transporter-2 (SGLT2) inhibitor; SU, sulfonylureas; TZD, thiazolinediones.

**FIGURE 2:**
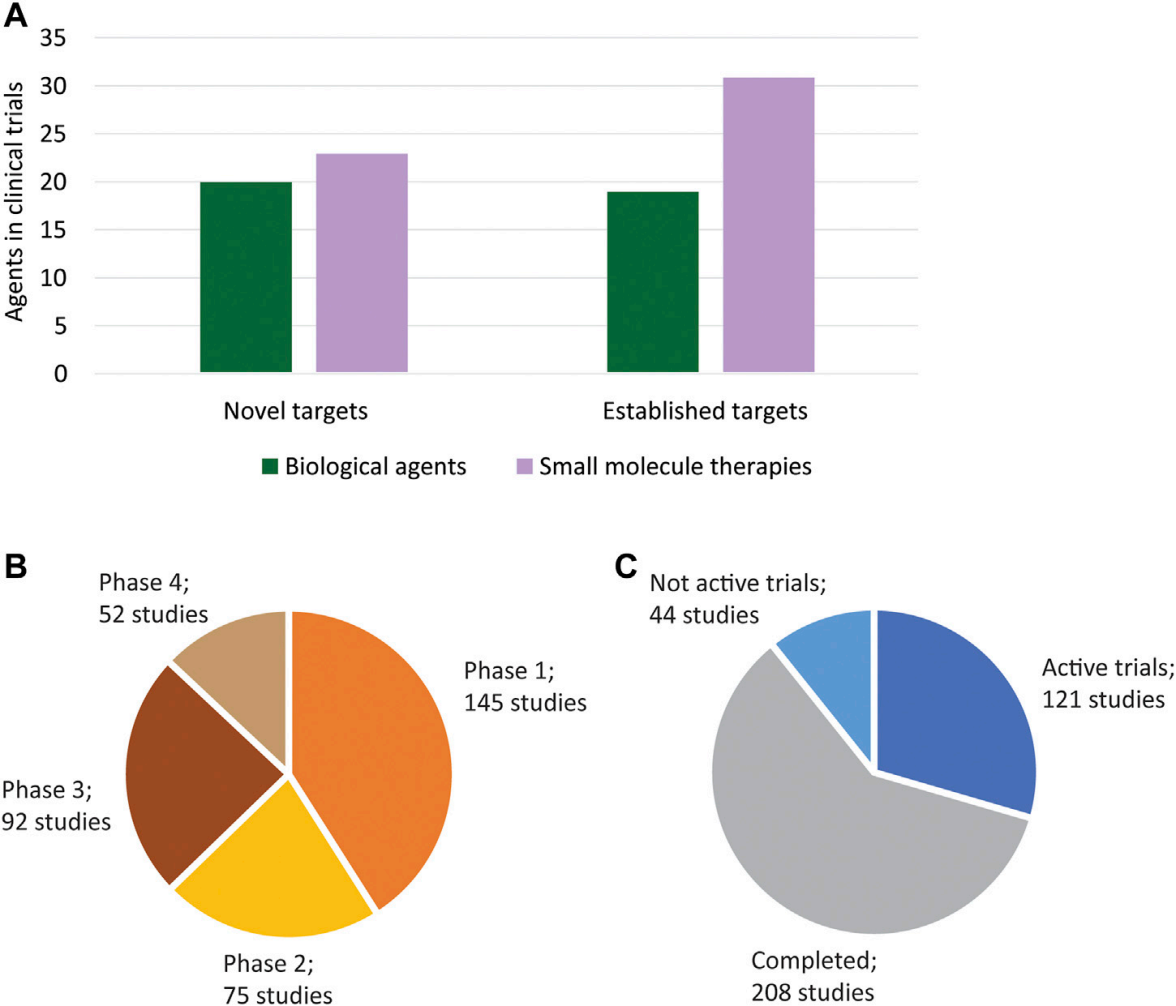
Agents in clinical development. **(A)** The ∼100 antihypertensive agents in clinical development include both biological agents and small molecule therapies. A high number of novel targets (i.e., targets that have not been validated through the approval an FDA-approval antidiabetic agent) are being investigated in clinical development. Novel targets include both single targets and unique combination regimens in trials. **(B)** ∼375 clinical studies associated with the investigative agents have been registered. A surprising number of agents are in phase 3 trials, indicating that more approvals may occur in the near future. Ten studies were not included as they did not have a phase identified. **(C)** The 121 active trials include registrations that have these statuses: *active, not recruiting; enrolling by invitation; not yet recruiting*; and *recruiting* as all of these are soon-to-be active. The 44 not active trials include studies with *suspended, terminated, unknown, and withdrawn* status.

In clinical development, nearly 100 antihyperglycemic agents are registered in ∼375 clinical trials from 2015 to 2020 (Figure 2). One-quarter of these agents are in phase III trials, with ten of them already having marketing approval from regulatory agencies in other countries and potentially seeking FDA approval. Thus, approximately half of the clinical agents target already established pathways, i.e., molecular targets that have been validated through the FDA approval of an agent targeting that pathway for the treatment of diabetes. However, a surprising number of the drugs in clinical trials have novel molecular targets, as monotherapies or combinations, that have not yet been validated through the approval of an agent by the FDA. A large proportion of clinical development is dedicated to new avenues for treating diabetes. Notably, at least six of these agents are in phase III trials, which indicates that potential first-in-class drugs for diabetes management may be approved soon.

## 3 Established Drug Classes for the Treatment of Diabetes

### 3.1 Types of Insulin

Different types of insulin comprise a considerable portion of FDA-approved drugs for diabetes treatment with at least 14 unique analogues and combination regimens. Insulin is perhaps one of the most studied proteins and has been an integral part of T2DM treatment. Recombinant insulin analogues have been developed that act in several different ways. Rapid-acting insulin analogues supply a bolus insulin level needed at mealtimes (prandial insulin) and include insulin lispro, aspart, and glulisine. Longer-acting insulins released slowly over a more extended period supply the basal insulin level needed throughout the day and night (basal insulin) and include detemir, glargine, and the ultra-long-acting degludec. Such a spectrum of insulin analogues enables combinations of different insulin forms, providing an effective basal-bolus therapy that more closely reflects physiological insulin secretion (Petersen and Shulman, 2018). In addition to insulin plus insulin combination regimens, insulin plus glucagon-like peptide-1 (GLP1) receptor agonist combinations have also been approved. Furthermore, basal insulin therapy combinations with other drugs or adjunctive therapies are also prescribed (Chaudhury et al., 2017). Even though insulin has been an important discovery for the treatment of diabetes, it is rarely used as a first-line treatment choice (van den Boom et al., 2020). Insulin administration comes with risks of developing severe hypoglycemia, cancer, and cardiovascular complications, and most often occurs when patients develop insulin tolerance and the dose administered has to be increased (Lebovitz, 2011).

### 3.2 Sulfonylureas (SU)

Until the approval of metformin, sulfonylureas (SU) were the only approved insulin competitors and were extensively used to treat T2DM. While currently, only three SU drugs are available for the prescription (glyburide, glipizide, and glimepiride). There have been at least four additional unique chemical SU compounds previously approved that are now discontinued (refer to Supplementary Table S2 for a full description of previously approved and now discontinued drugs for the T2DM treatment). Two SU and metformin combination regimens have been FDA-approved that are currently marketed: glyburide/metformin, glipizide/metformin. Combinations of glimepiride and metformin do exist, e.g., Amaryl M and Glimetal Lex, but are not approved by the FDA or the European Medicines Agency (Kim et al., 2014; AdisInsight, 2021). However, in the FDA approval of Amaryl (Glimepiride, 2021) it is stated that the two drugs can be taken together if monotherapies of each fail to work. The first SU compounds were discovered in the 1940s and entered the pharmaceutical market in the mid-1950s. The evolution of insulin therapies went in parallel with the search for alternative approaches. Among the first such attempts, compounds from the SU class were most interesting. Sulfonylureas mimic the effect of ATP in pancreatic beta cells and act as insulin-secreting agents (Tian et al., 1998; Al-Omary, 2017). SU molecules interact with the sulfonylurea receptors (SURs) on the surface of beta cells, which leads to inhibition of ATP-dependent inward-rectifier potassium ion channels (Ashcroft, 1996; Szewczyk, 1997; Liu et al., 2016). As a result, the intracellular concentration of potassium cations increases, leading to plasma membrane depolarization. These conditions stimulate the opening of voltage-gated calcium channels, and an increased concentration of cytosolic calcium cations leads to a surge in insulin secretion (Sulis et al., 2019).

SU drugs have been extensively prescribed to treat T2DM for more than 50 years. SUs are well-tolerated, and their popularity could be attributed to their low cost and the possibility of use as a monotherapy or in combination with metformin (Sola et al., 2015). SUs do not only interact with SURs in pancreatic beta cells, but also in smooth muscle cells and cardiac myocytes. This may explain why SU agents have been linked to a greater prevalence of hypoglycemia and cardiovascular risk (Rao et al., 2008; Schramm et al., 2011). However, most reports support the cardiovascular safety of SUs (Pop and Lingvay, 2017).

### 3.3 Biguanides

The approval of the biguanide metformin in 1995 significantly changed T2DM therapy and is the only FDA-approved antihyperglycemic agent in this drug class. Metformin selectively inhibits the mitochondrial isoform of glycerophosphate dehydrogenase, indirectly activates adenosine monophosphate-activated protein kinase (AMPK), and reduces cytosolic dihydroxyacetone phosphate while raising cytosolic NADH/NAD ratio (Musi et al., 2002; Wang et al., 2019). This results in decreased plasma glucose and lactate levels, reduced liver gluconeogenesis, hepatic glucose secretion, and endogenous glucose production (Foretz et al., 2014; Rena et al., 2017; Wang et al., 2019). Moreover, metformin can increase insulin sensitivity in muscle tissues. Currently, metformin is the only antihyperglycemic drug recommended by the American Diabetes Association and the European Association for the Study of Diabetes as initial oral therapy for patients with T2DM (Hostalek et al., 2015).

### 3.4 Alpha-Glucosidase Inhibitors

The first alpha-glucosidase inhibitor (AGI), acarbose, was approved by the FDA as an antihyperglycemic agent in 1995 and the second AGI, miglitol, followed in 1996. These are the only two AGIs approved for the United States market, although another AGI, voglibose, was approved by the Pharmaceuticals and Medical Devices Agency in Japan (Oki et al., 1999). In clinical development, the chewable tablet BTI-320 (PAZ320) recently completed a proof-of-concept study that showed low dose BTI-320 attenuated postprandial rise in blood glucose and reduced body weight modestly in pre-diabetic subjects. It is currently in the product pipeline at Boston Therapeutics, and due to the ease of administration and high levels of tolerance, it can be used as an adjunct to lifestyle modification for diabetes prevention (Trask et al., 2013).

Alpha-glucosidase is a widely expressed enzyme that cleaves glucosidic bonds. Inhibition of alpha-glycosidase prevents the digestion of complex carbohydrates to monosaccharides in the small intestine (Bischoff, 1994; Zhang et al., 2016). Thus, these agents act as pseudo-carbohydrates (substrate analogues), where they inhibit digestive enzymes and prevent oligo- and polysaccharides from being catabolized to monomers (Goto et al., 1995; Taira et al., 2000). This leads to less sugar being absorbed, resulting in lower postprandial glucose levels and reducing hyperglycemia (Lebovitz, 2011). AGIs have been shown to have similar efficacy as metformin, so they are often prescribed as a first-line treatment or combined with other antidiabetics. However, typical side effects of AGIs are flatulence, abdominal bloating and discomfort, and diarrhea. At the same time, a recent meta-analysis found that the use of AGI leads to an increase in liver transaminases, indicative of hepatotoxicity (Zhang et al., 2016).

### 3.5 Thiazolidinediones

Thiazolidinediones (TZDs) act as insulin sensitizers which activate peroxisome proliferator-activated receptors (PPARs), a broad family of nuclear receptors. The first TZD drug, troglitazone, was approved by the FDA in 1997; however, it was discontinued in 1999 due to severe hepatotoxicity. Currently, there are two marketed TZDs, rosiglitazone and pioglitazone, which were FDA-approved in 1999. TZD use has previously been limited due to concerns with safety issues and side effects. In addition, there was some controversy over cardiovascular toxicity with rosiglitazone and an increase in bladder cancer with pioglitazone. However, recent studies show no longer significant issues (see Lebovitz (2019) for review). Furthermore, the beneficial effects of TZDs on the cardiovascular risk factors associated with insulin resistance have been well documented. TZD drugs can be effective as a monotherapy or in a combination regimen. One combination regimen that consists of pioglitazone and metformin is currently marketed. Four TZD monotherapies and one combination with a dipeptidyl peptidase-4 (DPP4) inhibitor are in trials in clinical development. The most clinically advanced is lobeglitazone, which has already been approved in South Korea and is currently in phase III trials for additional combination treatments.

TZD molecules can interact with PPAR-α and PPAR-γ isoforms expressed primarily on fatty tissues and skeletal muscle. This leads to activating these receptors and stimulating complexation with another essential constituent–the retinoid X receptor. The triple complex can bind specifically to DNA by peroxisome proliferative response elements (PPRE) and act as a target gene promoter, thus stimulating gene expression (Yau et al., 2013). This therapeutic method leads to increased adiponectin levels, decreased gluconeogenesis, and increased glucose uptake in the muscle and fat. Adiponectin is a hormone secreted in adipose tissue that regulates glucose concentration by improving insulin sensitivity (Yu et al., 2002).

In general, prescription rates for SU and TZD drugs are experiencing a gradual decline (Kohro et al., 2013; Eibich et al., 2017; Wilkinson et al., 2018; Giorda et al., 2020). From 2000 to 2006, there was a rapid surge in TZD prescriptions (∼45%); however, most likely due to reports of safety issues, TZD use decreased (Wilkinson et al., 2018; Secrest et al., 2020). Currently, a guideline recommends a series of intensification steps to a baseline of initial metformin monotherapy in case of intolerance to metformin (Irons and Minze, 2014; American Diabetes Association, 2019). However, each country may manage the situation differently due to income levels, medicine development degrees, and population differences in physiology (Singla et al., 2019).

### 3.6 Incretin-Dependent Therapies (GLP1 Receptor Agonists and DPP4 Inhibitors)

In 2005 and 2006, the first incretin dependent T2DM therapies were approved, and they have become increasingly popular as monotherapies and in combination regimens since then. Incretin-depending treatments include glucagon-like peptide-1 (GLP1) mimetics which act as GLP1 receptor agonists and DPP4 inhibitors. Six injectable GLP1 receptor agonists were approved, including exenatide, liraglutide, dulaglutide, albiglutide, lixisenatide, and semaglutide. They differ in their lifetime in the bloodstream and in their ability to treat hyperglycemia (Yamamoto-Honda et al., 2018). Incretin therapies account for 30% of antidiabetic drugs in clinical development, with GLP1R agonists comprising the most significant proportion (20%). The clinical outcome of GLP1R agonists is robust, with 21 agents in clinical trials and the majority of them in phase I and II trials. Fifteen of these receptor agonists target just GLP1R, four agents also target the glucagon receptor (GCGR), one drug targets GLP1R plus GCGR plus the gastric inhibitory polypeptide receptor (GIPR), and one drug targets GLP1R and GIPR. The only GLP1R agonist in phase III trials is efpeglenatide from Hanmi Pharm; it demonstrated a dose-proportional pharmacokinetic profile, with an extended half-life and slow absorption in patients with T2DM (Sharma et al., 2018; Del Prato et al., 2020; Yoon et al., 2020).

There are currently four DPP4 inhibitors that have been FDA-approved: sitagliptin, saxagliptin, linagliptin, and alogliptin. However, at least seven additional DPP4 inhibitors have obtained approval from other regulating agencies and are currently registered in phase III and IV trials. It is not clear whether all these agents will seek FDA marketing approval or not. Also, there are ten DPP4 inhibitors in clinical development–two additional phase III drugs and one agent in phase II trials. The introduction of DPP4 inhibitors as antihyperglycemic agents brought significant changes in T2DM prescription trends. In Japan, for example, they were the most frequently prescribed drugs in the elderly in 2013 (49.1%) (Yamamoto-Honda et al., 2018).

GLP1 is one of the most crucial hormones in glucose metabolism and is released into the bloodstream by special L-cells in the ileum and colon. It is one of two significant incretins (intestinal secretion of insulin) hormones which stimulate insulin secretion and suppress glucagon synthesis (Meier et al., 2003; Andersen et al., 2018; Mathiesen et al., 2019). They act on the pancreas within 2–4 min and are rapidly inactivated afterwards. DPP4 selectively cleaves N-terminal dipeptides from proteins containing proline or alanine in the penultimate position, and GLP1 is its substrate (Röhrborn et al., 2015). Usually, this cleavage does not have any adverse effects on the glucose level–on the contrary–this inactivation is necessary. Nevertheless, in T2DM, the production of incretins is reduced, and their effects are weakened. Furthermore, the inactivation occurs so rapidly that there is not enough time for incretins to perform their physiological functions (Drucker and Nauck, 2006; Nauck and Meier, 2018). Therefore, this inactivation of incretins by DPP4, which is usually necessary, leads to hyperglycemia in T2DM patients. To overcome this, two possible mechanisms have been implemented: to create artificial long-acting GLP1 mimetics and to prevent the enzyme from cleaving its substrates. Both GLP1 receptor agonists and DPP4 inhibitors share some remarkable effects regarding beta-cell physiology in that they can improve beta-cell functioning and reduce apoptosis (Lee and Jun, 2014; Bugliani et al., 2018; Wang, et al., 2018; Jiang et al., 2020). However, this advantage may have a simultaneous drawback; with an increase in the survival of beta cells due to the reduced apoptosis, the potential risk of cancer increases. It is also worth mentioning that liraglutide and semiglutide, GLP1 receptor agonists, act as cardiovascular protectors (Marso et al., 2016; Bethel et al., 2018; Davies et al., 2018; Sharma et al., 2018; Husain et al., 2019; Husain et al., 2020). Moreover, the usage of GLP1 receptor agonists is associated with weight loss. At the same time, while DPP4 inhibitors do not influence weight gain, it is controversial whether or not they increase the risk of heart failure (Khalse and Bhargava, 2018; Sano, 2019).

### 3.7 Meglitinides

Two meglitinides have been FDA-approved: nateglinide in 2009 and repaglinide in 2013. Currently, there are no meglitinides in clinical trials. Meglitinides share a similar mechanism of action to sulfonylurea agents in that they increase insulin secretion in the pancreas. They bind to SURs in pancreatic beta cells but at a binding site different than SUs and induce the same reaction cascade that leads to insulin secretion (O’Brien et al., 2018). In contrast to SUs, meglitinides, nateglinide in particular, exhibit glucose-sensitive action whereby their potency increases at higher glucose concentrations (Hu et al., 2000). Meglitinides are short-acting and associated with lower hypoglycemia risks, weight gain, and chronic hyperinsulinemia than sulfonylurea drugs (Guardado-Mendoza et al., 2013). Other studies have since demonstrated that meglitinides could be associated with increased risk of hypoglycemia in diabetic patients with advanced chronic kidney disease (Wu et al., 2017).

### 3.8 Sodium-Glucose Cotransporter Type 2 Inhibitors

The most modern and promising drug class is SGLT2 inhibitors. The first SGLT2 inhibitors, canagliflozin, and dapagliflozin were approved in 2013, followed by additional monotherapy agents including empagliflozin in 2014 and ertugliflozin in 2017. Additionally, SGLT2 inhibitors are popular in combination regimens with metformin and DPP4 inhibitors and combinations of all three and TZD drugs. SGLT2 inhibitors are the second largest group of antidiabetic agents in clinical trials (12%) after incretin therapies. Three of the twelve drugs in phase II, III, and IV clinical trials have been previously approved by other regulating agencies. Five other agents are in phase III trials, indicating that new SGLT2 inhibitors may be approved soon.

Even though SGLT inhibitors reduce renal glucose reabsorption levels, which leads to glucose excretion (glucosuria) and weight loss (Taylor, et al., 2015; Lupsa and Inzucchi, 2018; Brown et al., 2019), they also appear to have good pharmacokinetic properties and are well tolerated (Ito et al., 2016). Moreover, this drug class has been shown to improve cardiovascular conditions in both diabetic and non-diabetic populations (Martens et al., 2017). Therefore SGLT-2 inhibitors have become the preferred glucose-lowering drugs to treat patients with T2DM at high risk of cardiovascular events, although it is also associated with urogenital infections (Wu et al., 2016; Kramer, et al., 2018). As a result, prescription rates for newer therapies, such as DPP4 and SGLT2 inhibitors, have grown. In the United Kingdom, in 2017, new prescription rates for DPP4 and SGLT2 inhibitors accounted for 42 and 22%, respectively (Eibich et al., 2017).

### 3.9 Drug Combinations

**FIGURE 3:**
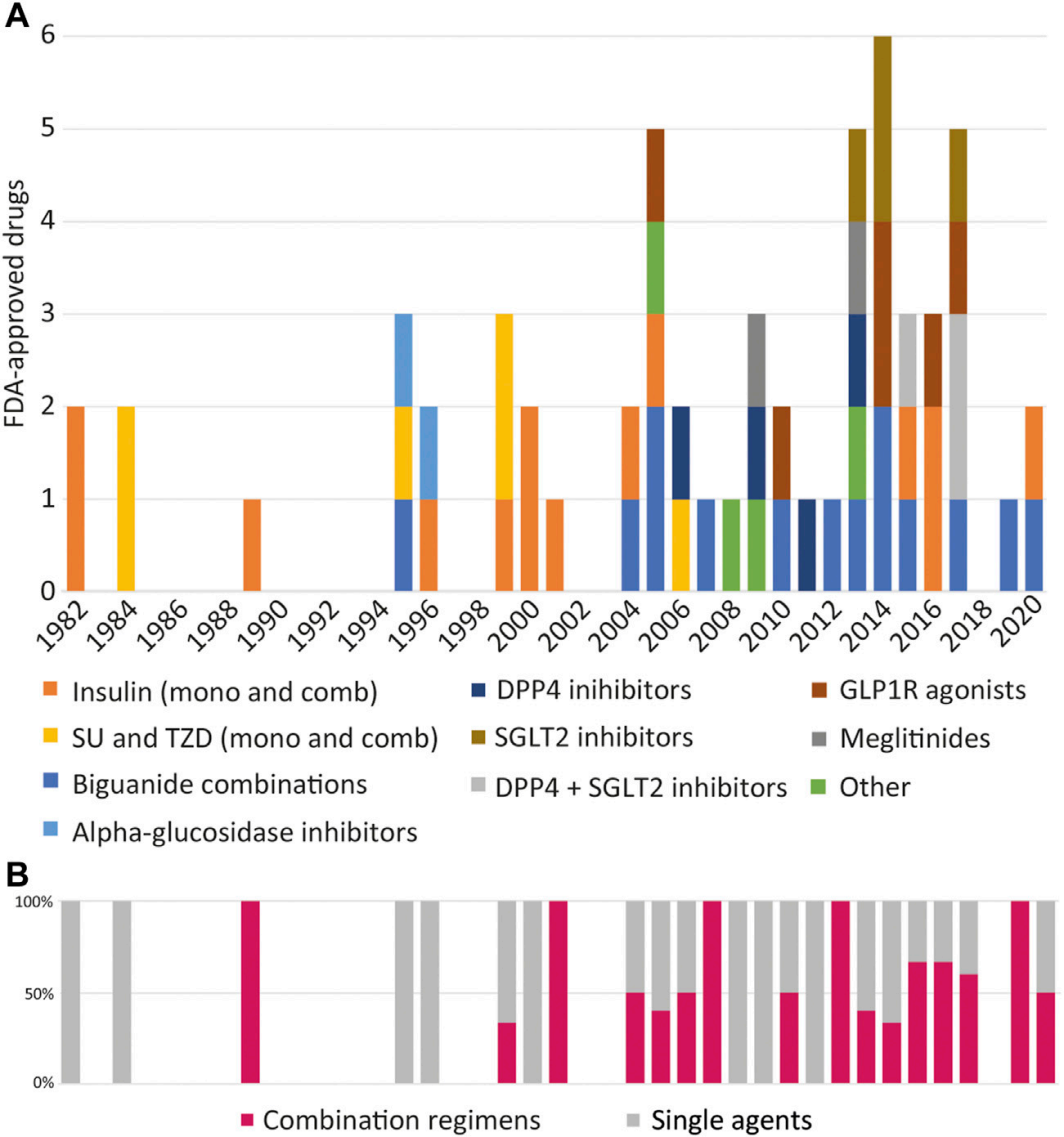
FDA-approved drug classes per year. **(A)** Timeline of the major classes of antihypertensive drugs that have approved including monotherapies (mono) and combinations (comb). DPP4, Dipeptidyl peptidase 4; GLP-1R, Glucagon-like peptide-1 (GLP-1) receptor; SGLT2, Sodium-glucose co-transporter-2; SU, Sulfonylureas; TZD, Thiazolinediones. **(B)** The proportion of combination regimens in comparison to monotherapies that have been approved each year. The use of agents in two and three combination regimens is increasing as treatment options expand to target multiple facets of diabetes pathophysiology.

The different types of approved oral combinations have steadily increased, along with the proportion of combinations being approved in comparison to monotherapies (Figures 3A,B). Nearly 40% of the approved antidiabetic drugs are combination regimens. FDA-approved combinations of antihyperglycemic drugs can be divided into two generations. First-generation combinations were mixtures of different insulin isoforms, where they differed in the method of preparation, natural source, duration of action, or concentrations. By 2004, the increase in the proportion of drug combinations began in earnest as second-generation antihyperglycemic combinations evolved. They consisted mainly of drugs that require oral administration, and in most cases, one of the components was metformin. To date, there are 23 unique antihyperglycemic drug combinations. The first triple combination regimen was approved in 2019, consisting of metformin, saxagliptin, and dapagliflozin. Another triple combination approval for metformin, linagliptin, and empagliflozin followed in 2020. This trend expounds on the idea that it is necessary to use all known approaches for the comprehensive treatment of T2DM.

Our analysis shows that most combinations that have been created are based on metformin. It also correlates well with global prescribing trends. Currently, metformin is the most optimal drug for monotherapy. Its prescription trends have dramatically increased over the past years, which cannot be said about other options for the initial therapy of T2DM. For instance, in the United Kingdom prescriptions for SUs and insulin declined by almost tenfold between 2000–2017 (Wilkinson et al., 2018; Ramzan et al., 2019). The 1998 United Kingdom Prospective Diabetes Study (UKPDS) shifted the trend toward using metformin instead of using SUs. Moreover, the introduction of long-acting insulins benefited patients with T2DM in the last stages of progression, often being the very last frontier to achieve glycemic control and help prevent chronic implications (Eibich et al., 2017).

## 4 Novel Drug Targets

**FIGURE 4:**
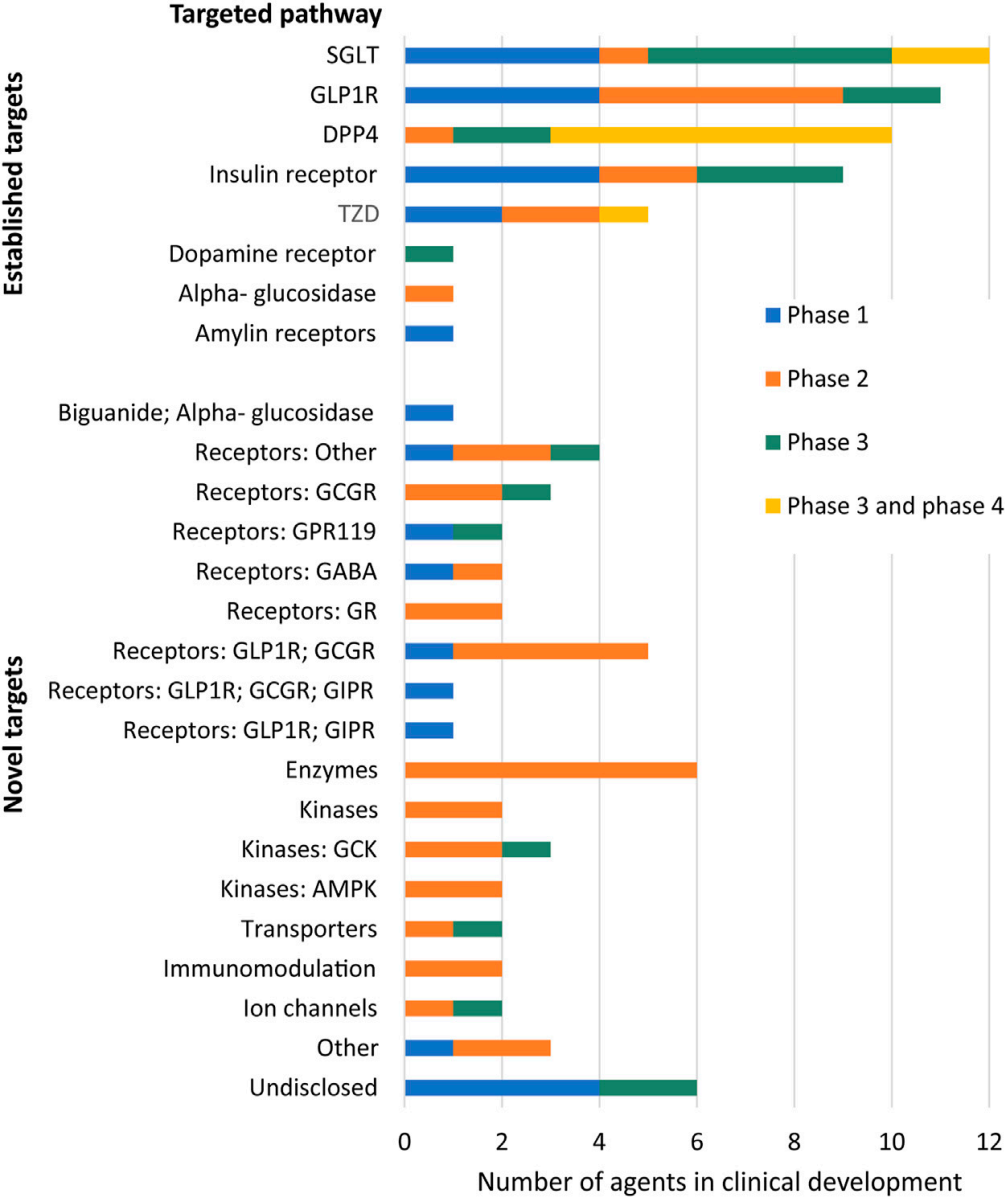
The molecular targets of the 99 anti-diabetic agents in clinical trials. The phase status is the highest clinical phase each agent has achieved. A surprising number clinical agents–nearly half–target novel molecular targets or combination regimens. Novel targets or pathways are those that have not yet been validated through approval of an FDA-approved drug for treatment of diabetes. Approximately half of the agents target already established pathways, i.e., molecular targets that have been validated through the FDA-approval of an agent targeting that pathway for the treatment of diabetes. Six of the agents had an undisclosed mechanism of action. DPP4, Dipeptidyl peptidase 4; GLP-1R, Glucagon-like peptide-1 (GLP-1) receptor; SGLT2, Sodium-glucose co-transporter-2; TZD, Thiazolinediones; GCGR, Glucagon receptor; GPR119, Glucose-dependent insulinotropic receptor (G-Protein coupled receptor 119); GR, Glucocorticoid receptor; GIPR, Gastric Inhibitory Polypeptide Receptor; GCK, glucokinase; AMPK, 5′-AMP-activated protein kinase.

More than 40% of the agents identified in clinical trials target novel therapeutic molecules or combinations of targets (Figure 4). Receptors and enzymes and the largest classes of novel targets, followed by transporters and ion channels. Several key targets are described in the following section.

### 4.1 Receptors

Glucose protein-coupled receptor 119 (GPR119) plays a critical role in glucose homeostasis and is expressed in pancreatic β-cells and enteroendocrine cells (Fredriksson et al., 2003; Neelamkavil et al., 2018). Previous studies have shown that agonism of GPR112 stimulates insulin and incretin secretion (Semple et al., 2008; Kang, 2013; Ritter et al., 2016). Thus, the mechanism of action resembles the incretin effect in glucose metabolism. Furthermore, animal studies suggest that agonism of GPR119 has favorable outcomes regarding weight gain and food intake (Overton et al., 2008). However, due to the chemistry of GPR119 agonists thus far, unintended side effects may occur and may have been a contributing factor in the discontinuation of previous clinical agents (Ritter et al., 2016). The second-generation GPR119 agonist DA-1241 is currently in phase I trials and will hopefully provide diabetic patients with significant glucose-lowering benefits and better safety profiles (Kang, 2013; Ritter et al., 2016).

The gastric inhibitory polypeptide (GIP) is an incretin hormone that works similarly to GLP1 in stimulating pancreatic β cells for insulin secretion in a glucose-dependent manner. Tirzepatide is currently active in clinical trials (Phase III), which acts as a dual agonist. Binding to both the GLP1 receptor and GIP receptor, a study with GIP showed decreased body weight and hemoglobin A1c (Mathiesen et al., 2019).

Thyroid hormone receptors (THR) have typically been targeted in the treatment of metabolic disorders, but they have also been shown to be attractive targets in the treatment of diabetes (Saponaro et al., 2020). THR agonism studies in mice resulted in increased energy expenditure, increased insulin sensitivity, and lowered glucose concentrations. These effects may be because THR is highly expressed in the liver, skeletal muscles, and kidneys (Lin and Sun, 2011; Zambad et al., 2011). There is one thyroid hormone receptor agonist (TRC150094), which is a functional analogue of iodothyronines. It is currently active in phase III clinical trials to evaluate the safety in lowering cardiovascular risk in patients with diabetes.

### 4.2 Enzymes–General

Diacylglycerol acyltransferase (DGAT) is a crucial enzyme in triacylglycerol (TAG) synthesis, which catalyzes the final dedicated step of TAG synthesis. DGAT deficient mice showed a significant reduction in the postprandial increase of plasma TAG and were resistant to diet-induced obesity due to increased energy expenditure. Even more, the DGAT1 knockout mice had enhanced insulin sensitivity. However, increased activity of DGAT in T2DM patients can cause beta-cell dysfunction. Therefore, selective DGAT inhibitors have been designed to manage metabolic diseases such as obesity and T2DM due to their ability to prevent abnormal TAG levels and beta-cell damage. Currently, at least one promising agent (IONIS DGAT2Rx) from this drug group has completed phase II clinical trials. Being a hepatoprotectant, it is not a classic hyperglycemic drug, but it indirectly affects glycemia by increasing insulin sensitivity and protecting islet β-cells (Zhu et al., 2019; Hong et al., 2020).

The kallikrein-kinin system may also provide an exciting approach for diabetes treatment. It has been shown that bradykinin (BK) resulted in increased glucose uptake and insulin sensitivity through the bradykinin type 2 receptor (BK2R) signaling (Lau et al., 2020). In addition, studies on diabetic mice models treated with human tissue kallikrein 1 gene resulted in lowered glucose concentration, controlled hypoglycemia, and reversed insulin resistance (Kolodka et al., 2014). This therapeutic strategy is being investigated with DM-199, a recombinant human tissue kallikrein-1 protein currently in phase II trials to treat diabetes.

Fructose-1,6-bisphosphatase-1 (FBP1), an enzyme involved in gluconeogenesis, is another constituent in glucose homeostasis (Zhao et al., 2018). An unmet issue in T2DM pathology is the endogenous glucose production in the liver, which may be mediated by inhibiting FBP1. While metformin can indirectly mediate this activity (Hunter et al., 2018), the investigative agent VK 0612 directly inhibits FBP1 and has completed phase II clinical trials.

Methionine aminopeptidase 2 (MetAP2) is an enzyme that cleaves off methionine from the N-terminus of new proteins. It has been shown that MetAP2 has a crucial role in angiogenesis, which is why MetAp2 inhibitors have been used in cancer treatment (Chun et al., 2005; Morgen et al., 2016). However, recent studies have shown that inhibition of MetAP2 is a strong candidate for treating obesity and diabetes (Joharapurkar et al., 2014). Inhibition of MetAP2 induced clinically meaningful reductions in blood glucose and increased weight loss (Siddik et al., 2019; Wentworth et al., 2020). The clinical agent ZGN-1061 is a MetAP2 inhibitor with promising results with improved glucose control and lowered weight in preclinical studies and a recently completed phase II clinical trial (Burkey et al., 2018; Wentworth et al., 2020).

Angiopoietin-related protein 3 (ANGPTL3) is another protein involved in angiogenesis and hence cancer; however, it also has essential functions in glucose metabolism. Its principal function is inhibiting lipoprotein lipase and controlling triglyceride levels in plasma (Lupo and Ferri, 2018; Ahmad et al., 2019; Lang and Frishman, 2019). How exactly ANGPTL3 functions in glucose metabolism is unclear. However, it is speculated that it lowers insulin sensitivity and increases insulin resistance by increasing free fatty acids (Christopoulou et al., 2019). Thus, inhibition of ANGPTL3 is a promising strategy and a potential drug target for T2DM and other metabolic disorders. ISIS-703802, a designed ANGPTL3 inhibitor, recently completed phase II trials in subjects with hypertriglyceridemia, T2DM, and non-alcoholic fatty liver disease (Jiang et al., 2019).

### 4.2.1 Enzymes–Kinases

Glucokinase is a crucial enzyme that maintains normal glucose homeostasis and has a glucostatic effect in the blood because it initiates gluconeogenesis. Activating glucokinase by small molecule therapeutics may increase insulin secretion from the pancreas, promote glycogen synthesis in the liver, and thus reduce hepatic glucose output (Toulis et al., 2020). Therefore, a promising strategy for antihyperglycemic drugs is through glucokinase activators (GKA). Research and development began in the early 1990s, and preclinical animal studies showed that GKAs effectively normalize blood glucose levels; however, the activation leads to severe hyperlipidemia, vascular hypertension, and other negative consequences (Matschinsky, 2013). Hence many GKAs have entered trials (e.g., Piragliatin, ARRY-403, AZD1656, PSN010), although they have subsequently discontinued clinical progression. However, there has been renewed interest due to the development of several next-generation GKAs: dorzagliatin, a novel, dual-acting agent that targets both pancreatic and hepatic glucokinases, and TTP399, a hepatoselective compound (Toulis et al., 2020). Dorzaliatin is currently in phase III trials, and TTP339 and SY004, another new GKA, are in phase II trials.

AMP-activated protein kinase (AMPK) is an enzyme for energy regulation. When energy levels are low, it promotes glucose update in skeletal muscles and reduces gluconeogenesis. It was found that a healthy lifestyle that includes calorie restriction, exercise, and hormones that encourage longevity like leptin and adiponectin activate AMPK. In addition, some antihyperglycemic drugs, such as metformin or canagliflozin, can indirectly activate AMPK via regulating cation transporters (Coughlan et al., 2014; Steinberg and Carling, 2019). Currently, direct activators of AMPK have been designed, and several are registered in trials: PXL770 and PBI-4050 have recently completed phase II studies.

Fructokinase (FK) is an enzyme in the liver, intestine, and kidney cortex that converts fructose into fructose-1-phosphate. Since fructokinase lacks a negative feedback system, its activation leads to the depletion of phosphates, thus leading to activation of AMP deaminase and the formation of uric acid, which causes inflammation in the cells. This inflammation happens in pancreatic islets and can lead to insulin resistance (Khitan and Kim, 2013). Thus, inhibition of this pathway could be an exciting approach in the treatment of hyperglycemia. The fructokinase inhibitor, PF-06835919, is currently in phase II clinical trials.

Tolimidone (MLR-1023) has completed phase II in patients with uncontrolled T2DM and is currently active in phase II with patients treated with metformin. It is a potent Lyn protein tyrosine kinase stimulant (Ochman et al., 2012; Saporito et al., 2012). Lyn tyrosine kinase is vital for insulin sensitivity and glucose metabolism. It phosphorylates insulin receptor substrates which attenuate insulin receptor signaling (Lee et al., 2020; Lipinski and Reaume, 2020). It was shown to lower blood glucose similarly to metformin in preclinical studies without a hypoglycemic episode. In addition, tolimidone is insulin-dependent and was shown to increase insulin sensitivity (Ochman et al., 2012).

### 4.3 Transporters

Another attractive agent is MSDC-0602K which acts as a mitochondrial membrane transport protein modulator that increases insulin sensitivity. Impaired mitochondrial function has been previously connected with the development of diabetes and its complications (Sivitz and Yorek, 2010). MSDC-0602K is the second generation of insulin sensitizers expected to have fewer side effects than the first-generation compounds. It is planned to start phase III clinical trials in 2022 (Harrison et al., 2020).

### 4.4 Targets Not Currently Active in Clinical Development

11-beta-hydroxysteroid dehydrogenase 1 (EC 1.1.1.146) converts cortisone to cortisol, thus indirectly increasing glucose output in the liver via the ability of cortisol to activate corticoid receptors and transcription of phosphoenolpyruvate kinase. It was estimated that 11β-HSD1 activity increases in patients with T2DM, and hence why synthesizing potent inhibitors has looked promising. Several agents (e.g., Poxel, CNX-010, SAR-184841) have reached preclinical studies or early research phases, but there were no recent reports of further development. At least one 11β-HSD1 inhibitor, INCB13739, has completed phase II in clinical trials as an antihyperglycemic agent, but neither statements of development nor discontinuation were identified (Paranjeet et al., 2018; Shukla et al., 2019).

Increased cannabinoid-1 (CB1) receptor activity can cause obesity and obesity-related T2DM; peripheral effects of CB1 antagonism are decreased bodyweight, improved glucose tolerance, increased adiponectin, and decreased insulin resistance. All of these consequences are favorable in regards to the treatment of hyperglycemia. However, CB1 antagonism in the central nervous system increases anxiety and depression (Lu et al., 2016). The first cannabinoid-1 receptor antagonist, rimonabant, initially approved in Europe for T2DM treatment, was subsequently withdrawn due to the risk of severe mood disorders (Williams et al., 2020). Another antagonist, Tetrahydrocannabivarin-9, has completed phase II in clinical trials but has not clinically progressed (Jadoon et al., 2016).

## 5 Discussion

Here we have provided a comprehensive study of the clinical trials and FDA approved drugs in relationship to their therapeutic targets. It is clear that the number of therapeutic alternatives to treat T2DM are increasing and now there are nearly 60 drugs approved by the FDA. Beyond this there are nearly 100 additional antihyperglycemic agents being evaluated in clinical trials. In addition to the standard treatments of insulin therapy and metformin, there are new drug combinations, e.g., containing metformin, SGLT2 inhibitors and DPP4 inhibitors, that have gained substantial use during the last decade. Furthermore, there are several interesting alternatives, such as lobeglitazone, efpeglenatide and tirzepatide, in ongoing clinical trials.

Major advancements within diabetes research have been made since the ground-breaking discovery of insulin in the early 1920s (Quianzon and Cheikh, 2012). Today’s antihyperglycemic drugs target a variety of pathological mechanisms implicated in T2DM, ranging from insulin secretion (e.g., SUs), peripheral glucose uptake (e.g., biguanides) and glucose reabsorption (e.g., SGLT2 inhibitors) (Chaudhury et al., 2017). Although insulin analogues remain a reliable approach to treat late stage T2DM, insulin therapy is no longer used in the initial stages of the disease. Over the past decades, the number of approved insulin analogues on the pharmaceutical market has remained relatively constant. However, the FDA recently withdrew approval of two insulin combinations (Ryzodeg was discontinued in 2018, and Novolog Mix 50/50 in 2019); while novel combinations containing insulins and GLP1R agonists have been approved, such as Xultophy (insulin degludec/liraglutide) and Soliqua (insulin glargine/lixisenatide). Since 1995, metformin has become the leading antihyperglycemic agent in the initial stages of T2DM and in combination with other drugs in the later stages (13 out of 24 drug combinations approved by the FDA contain metformin). Recent approvals have been made for triple drug combinations containing metformin and other modern antihyperglycemic drugs, including four unique formulae with SGLT2is and two metformin/SGLT2i/DPP4is. These novel combinations are Qternmet XR (metformin/saxagliptin/dapagliflozin, approved in 2019) and Trijardy XR (metformin/linagliptin/empagliflozin, approved in 2020). These triple combinations reinforce the treatment paradigm of comprehensively targeting multiple pathways for T2DM management.

The countries with the largest diabetic populations are China (140.9 million diabetics), India (74.2 million diabetics) and the United States (32.2 million diabetics) (International Diabetes Federation, 2021). After insulin, oral hypoglycemic agents account for the majority of sales on the Chinese antidiabetic market, while injectable GLP1R agonists dominate anti-diabetic drug sales globally (Zhang, 2018). The GLP1R agonists Ozempic (semaglutide), by Novo Nordisk, and Trulicity (dulaglutide), by Eli Lilly, have anticipated sales values of 6.2 and 6.6 billion USD, respectively in 2024 (Statista, 2021). Available prescription data also indicate an increasing preference for additional modern drugs, such as DPP4- and SGLT2 inhibitors. At present, there are at least 19 more GLP1R agonists, 10 DPP4- and 12 SGLT2-inhibitors in clinical trials, and it may be expected that more drugs from these therapeutic classes will appear on the pharmaceutical market soon. These drugs are highly effective, but they are more commonly prescribed to patients in economically developed countries, e.g., United States and Canada, due to their high cost. In both China and India, metformin is the most preferred first-line drug across the lifetime duration of diabetes (Hu and Jia, 2018; Singla et al., 2019). The emergence of metformin has influenced a decrease in the prescription trends of SUs and TZDs in economically developed countries (Srinivasan et al., 2018). Similar trends are observed in developing countries, but more often, less expensive drugs, such as SUs, are prescribed as add-ons to metformin (Mohan et al., 2020). However, the DPP4 inhibitors are catching up with SUs as second-line treatment after metformin in India, while SGLT2 inhibitors are being used as third- or fourth-line antidiabetic drugs (Singla et al., 2019). North America is projected to continue to hold a significant number of shares in the antidiabetic drug market (Knowledge Sourcing Intelligence LLP, 2021). This is largely attributed to both the high prevalence of diabetes and the dominating presence of pharmaceutical companies within this region (Knowledge Sourcing Intelligence LLP, 2021). However, Asia-Pacific regions are experiencing rising sales of over the counter antidiabetic drugs, which are both less expensive and more accessible than prescription anti-diabetics (Mohan et al., 2020; Mordor Intelligence, 2021). As T2DM diagnoses are increasing globally, it is essential to establish cost-effective disease management across both developing and developed countries.

Even though the T2DM topic has been well-reviewed, there is a continued need to improve treatment strategies and to investigate possible drug targets. This has become even more pressing during the COVID-19 pandemic, where infection with the SARS-CoV-2 virus has been described to both trigger the onset of diabetes and contribute to pre-existing diabetic complications (Hollstein et al., 2020; Marchand et al., 2020; Wu et al., 2021). The clinical course of COVID-19 may also be modulated by the type of antidiabetic drug the patient is receiving, as SGLT2 inhibitors and GLP1R agonists could exacerbate the infection while DPP4 inhibitors may act as mitigators (Mirabelli et al., 2020). The large heterogeneity of T2DM is also creating a push to personalize T2DM treatments to achieve an optimal therapeutic response, while minimizing adverse effects for each patient. To this end, characteristics such as patient compliance, ease of administration, weight gain, and low risk of hypoglycemia are increasingly being considered beyond just the tolerability and efficacy of the anti-diabetics (Miller et al., 2014). Integrated personalized diabetes management, incorporating the patient’s attitude, medical history and social support, has been highly successful in maintaining glycemic control, increasing patient adherence and overall treatment satisfaction in large scale randomized controlled studies (Kulzer et al., 2018). Moreover, combining antihyperglycemic drugs of different classes may counteract the adverse effects of each other, thus enhancing their efficacy (Pappachan et al., 2019). Another ongoing pursuit is the development of oral insulin administration, which would provide a more convenient delivery of insulin in comparison to subcutaneous injection. The oral insulin capsule ORMD-0801, by Oramed, showed promising results in Phase II trials and T2DM patients are currently being enrolled for Phase III to further evaluate the drug’s future potential (Eldor et al., 2021).

In conclusion, there has been major progress in T2DM pharmacological therapy during the last decade. The rapid pace in which diabetology is developing makes it challenging to keep up with the interesting and innovative therapeutic approaches currently used. Therefore, we considered it necessary to compile up to date antihyperglycemic drugs approved by the FDA and explore recent data on new potential antidiabetic agents. Our review provides key points regarding each significant class of antihyperglycemic drugs, gives insight into which treatment options have been successful, and the novel mechanisms currently explored in clinical development.

## References

[B1] Ahmad Z. Banerjee P. Hamon S. Chan K. C. Bouzelmat A. Sasiela W. J. (2019). Inhibition of Angiopoietin-like Protein 3 with a Monoclonal Antibody Reduces Triglycerides in Hypertriglyceridemia. Circulation 140 (6), 470–486. 10.1161/CIRCULATIONAHA.118.039107 31242752 PMC6686956

[B2] Al-Omary F. A. M. (2017). Gliclazide. Profiles Drug Subst. Excip Relat. Methodol. 42, 125–192. 10.1016/bs.podrm.2017.02.003 28431776

[B3] Andersen A. Lund A. Knop F. K. Vilsbøll T. (2018). Glucagon-like Peptide 1 in Health and Disease. Nat. Rev. Endocrinol. 14 (7), 390–403. 10.1038/s41574-018-0016-2 29728598

[B4] Artasensi A. Pedretti A. Vistoli G. Fumagalli L. (2020). Type 2 Diabetes Mellitus: A Review of Multi-Target Drugs. Molecules 25 (8). 10.3390/molecules25081987 PMC7221535 32340373

[B5] Ashcroft F. M. (1996). Mechanisms of the Glycaemic Effects of Sulfonylureas. Horm. Metab. Res. 28 (9), 456–463. 10.1055/s-2007-979837 8911983

[B6] Association, American Diabetes (2019). 9. Pharmacologic Approaches to Glycemic Treatment: Standards of Medical Care in Diabetes-2019. Diabetes Care 42 (Suppl. 1), S90–S102. 10.2337/dc19-S009 30559235

[B7] Attwood M. M. Jonsson J. Rask-Andersen M. Schiöth H. B. (2020). Soluble Ligands as Drug Targets. Nat. Rev. Drug Discov. 19, 695–710. 10.1038/s41573-020-0078-4 32873970

[B8] Attwood M. M. Rask-Andersen M. Schiöth H. B. (2018). Orphan Drugs and Their Impact on Pharmaceutical Development: (Trends in Pharmacological Sciences 39, 525-535, 2018). Trends Pharmacol. Sci. 39 (12), 1077. 10.1016/j.tips.2018.09.007 29779531

[B9] Attwood M. M. Fabbro D. Sokolov A. V. Knapp S. Schiöth H. B. (2021). Trends in Kinase Drug Discovery: Targets, Indications and Inhibitor Design. Nat. Rev. Drug Discov. 20, 839–861. 10.1038/s41573-021-00252-y 34354255

[B10] Bethel M. A. Patel R. A. Merrill P. Lokhnygina Y. Buse J. B. Mentz R. J. EXSCEL Study Group (2018). Cardiovascular Outcomes with Glucagon-like Peptide-1 Receptor Agonists in Patients with Type 2 Diabetes: a Meta-Analysis. Lancet Diabetes Endocrinol. 6 (2), 105–113. 10.1016/S2213-8587(17)30412-6 29221659

[B11] Bischoff H. (1994). Pharmacology of Alpha-Glucosidase Inhibition. Eur. J. Clin. Invest. 24 Suppl 3 (Suppl. 3), 3–10. 10.3406/galim.1994.1263 8001624

[B12] Brown E. Wilding J. P. H. Barber T. M. Alam U. Cuthbertson D. J. (2019). Weight Loss Variability with SGLT2 Inhibitors and GLP-1 Receptor Agonists in Type 2 Diabetes Mellitus and Obesity: Mechanistic Possibilities. Obes. Rev. 20 (6), 816–828. 10.1111/obr.12841 30972878

[B13] Bugliani M. Syed F. Paula F. M. M. Omar B. A. Suleiman M. Mossuto S. (2018). DPP-4 Is Expressed in Human Pancreatic Beta Cells and its Direct Inhibition Improves Beta Cell Function and Survival in Type 2 Diabetes. Mol. Cel Endocrinol 473, 186–193. 10.1016/j.mce.2018.01.019 29409957

[B14] Burkey B. F. Hoglen N. C. Inskeep P. Wyman M. Hughes T. E. Vath J. E. (2018). Preclinical Efficacy and Safety of the Novel Antidiabetic, Antiobesity MetAP2 Inhibitor ZGN-1061. J. Pharmacol. Exp. Ther. 365 (2), 301–313. 10.1124/jpet.117.246272 29491038

[B15] Chaudhury A. Duvoor C. Reddy Dendi V. S. Kraleti S. Chada A. Ravilla R. (2017). Clinical Review of Antidiabetic Drugs: Implications for Type 2 Diabetes Mellitus Management. Front. Endocrinol. (Lausanne) 8, 6. 10.3389/fendo.2017.00006 28167928 PMC5256065

[B16] Chawla A. Chawla R. Jaggi S. (2016). Microvasular and Macrovascular Complications in Diabetes Mellitus: Distinct or Continuum? Indian J. Endocrinol. Metab. 20 (4), 546–551. 10.4103/2230-8210.183480 27366724 PMC4911847

[B17] Chikara G. Sharma P. K. Dwivedi P. Charan J. Ambwani S. Singh S. (2018). A Narrative Review of Potential Future Antidiabetic Drugs: Should We Expect More? Indian J. Clin. Biochem. 33 (2), 121–131. 10.1007/s12291-017-0668-z 29651202 PMC5891460

[B18] Christopoulou E. Elisaf M. Filippatos T. (20192019). Effects of Angiopoietin-like 3 on Triglyceride Regulation, Glucose Homeostasis, and Diabetes. Dis. Markers 2019, 6578327. 10.1155/2019/6578327 PMC6421734 30944669

[B19] Chun E. Han C. K. Yoon J. H. Sim T. B. Kim Y. K. Lee K. Y. (2005). Novel Inhibitors Targeted to Methionine Aminopeptidase 2 (MetAP2) Strongly Inhibit the Growth of Cancers in Xenografted Nude Model. Int. J. Cancer 114 (1), 124–130. 10.1002/ijc.20687 15523682

[B20] Coughlan K. A. Valentine R. J. Ruderman N. B. Saha A. K. (2014). AMPK Activation: a Therapeutic Target for Type 2 Diabetes? Diabetes Metab. Syndr. Obes. 7, 241–253. 10.2147/DMSO.S43731 25018645 PMC4075959

[B21] Davies M. J. Aronne L. J. Caterson I. D. Thomsen A. B. Jacobsen P. B. Marso S. P. (2018). Liraglutide and Cardiovascular Outcomes in Adults with Overweight or Obesity: Apost Hoc Analysis from SCALE Randomized Controlled trialsLiraglutide and Cardiovascular Outcomes in Adults with Overweight or Obesity: A Post Hoc Analysis from SCALE Randomized Controlled Trials. Diabetes Obes. Metab. 20 (3), 734–739. 10.1111/dom.13125 28950422 PMC5836948

[B22] Del Prato S. Kang J. Trautmann M. E. Stewart J. Sorli C. H. Derwahl M. (2020). Efficacy and Safety of Once-Monthly Efpeglenatide in Patients with Type 2 Diabetes: Results of a Phase 2 Placebo-Controlled, 16-week Randomized Dose-Finding Study. Diabetes Obes. Metab. 22 (7), 1176–1186. 10.1111/dom.14020 32128957 PMC7383886

[B23] Drucker D. J. Nauck M. A. (2006). The Incretin System: Glucagon-like Peptide-1 Receptor Agonists and Dipeptidyl Peptidase-4 Inhibitors in Type 2 Diabetes. Lancet 368 (9548), 1696–1705. 10.1016/S0140-6736(06)69705-5 17098089

[B24] Eibich P. Green A. Hattersley A. T. Jennison C. Lonergan M. Pearson E. R. (2017). Costs and Treatment Pathways for Type 2 Diabetes in the UK: A Mastermind Cohort Study. Diabetes Ther. 8 (5), 1031–1045. 10.1007/s13300-017-0296-x 28879529 PMC5630552

[B25] Eldor R. Neutel J. Homer K. Kidron M. (2021). Efficacy and Safety of 28-day Treatment with Oral Insulin (ORMD-0801) in Patients with Type 2 Diabetes: A Randomized, Placebo-Controlled Trial. Diabetes Obes. Metab. 23 (11), 2529–2538. 10.1111/dom.14499 34310011

[B26] Foretz M. Guigas B. Bertrand L. Pollak M. Viollet B. (2014). Metformin: from Mechanisms of Action to Therapies. Cell Metab 20 (6), 953–966. 10.1016/j.cmet.2014.09.018 25456737

[B27] Fredriksson R. Höglund P. J. Gloriam D. E. Lagerström M. C. Schiöth H. B. (2003). Seven Evolutionarily Conserved Human Rhodopsin G Protein-Coupled Receptors Lacking Close Relatives. FEBS Lett. 554 (3), 381–388. 10.1016/s0014-5793(03)01196-7 14623098

[B28] Giorda C. B. Orsi E. De Cosmo S. Bossi A. C. Guerzoni C. Cercone S. (2020). Prescription of Sulphonylureas Among Patients with Type 2 Diabetes Mellitus in Italy: Results from the Retrospective, Observational Multicentre Cross-Sectional SUSCIPE (Sulphonyl_UreaS_Correct_Internal_Prescription_Evaluation) Study. Diabetes Ther. 11 (9), 2105–2119. 10.1007/s13300-020-00871-5 32734558 PMC7434823

[B29] Glimepiride (2021). Glimepiride/metformin - Laboratorios Silanes - AdisInsight. Available at: https://adisinsight.springer.com/drugs/800045426 (Accessed December 27, 2021).

[B30] Goto Y. Yamada K. Ohyama T. Matsuo T. Odaka H. Ikeda H. (1995). An Alpha-Glucosidase Inhibitor, AO-128, Retards Carbohydrate Absorption in Rats and Humans. Diabetes Res. Clin. Pract. 28 (2), 81–87. 10.1016/0168-8227(95)01065-l 7587923

[B31] Gourgari E. Wilhelm E. E. Hassanzadeh H. Aroda V. R. Shoulson I. (2017). A Comprehensive Review of the FDA-Approved Labels of Diabetes Drugs: Indications, Safety, and Emerging Cardiovascular Safety Data. J. Diabetes Complications 31 (12), 1719–1727. 10.1016/j.jdiacomp.2017.08.005 28939018

[B32] Guardado-Mendoza R. Prioletta A. Jiménez-Ceja L. M. Sosale A. Folli F. (2013). The Role of Nateglinide and Repaglinide, Derivatives of Meglitinide, in the Treatment of Type 2 Diabetes Mellitus. Arch. Med. Sci. 9 (5), 936–943. 10.5114/aoms.2013.34991 24273582 PMC3832818

[B33] Halban P. A. Polonsky K. S. Bowden D. W. Hawkins M. A. Ling C. Mather K. J. (2014). β-Cell Failure in Type 2 Diabetes: Postulated Mechanisms and Prospects for Prevention and Treatment. Diabetes Care 37 (6), 1751–1758. 10.1210/jc.2014-142510.2337/dc14-0396 24812433 PMC4179518

[B34] Harrison S. A. Alkhouri N. Davison B. A. Sanyal A. Edwards C. Colca J. R. (2020). Insulin Sensitizer MSDC-0602K in Non-alcoholic Steatohepatitis: A Randomized, Double-Blind, Placebo-Controlled Phase IIb Study. J. Hepatol. 72 (4), 613–626. 10.1016/j.jhep.2019.10.023 31697972

[B35] Hauser A. S. Attwood M. M. Rask-AndersenSchiöth M. H. B. Schiöth H. B. Gloriam D. E. (2017). Trends in GPCR Drug Discovery: New Agents, Targets and Indications. Nat. Rev. Drug Discov. 16, 829–842. 10.1038/nrd.2017.178 29075003 PMC6882681

[B36] Hollstein T. Schulte D. M. Schulz J. Glück A. Ziegler A. G. Bonifacio E. (2020). Autoantibody-negative Insulin-dependent Diabetes Mellitus after SARS-CoV-2 Infection: a Case Report. Nat. Metab. 2 (10), 1021–1024. 10.1038/s42255-020-00281-8 32879473

[B37] Hong D. J. Jung S. H. Kim J. Jung D. Ahn Y. G. Suh K. H. (2020). Synthesis and Biological Evaluation of Novel Thienopyrimidine Derivatives as Diacylglycerol Acyltransferase 1 (DGAT-1) Inhibitors. J. Enzyme Inhib. Med. Chem. 35 (1), 227–234. 10.1080/14756366.2019.1693555 31752563 PMC6882492

[B38] Hostalek U. Gwilt M. Hildemann S. (2015). Therapeutic Use of Metformin in Prediabetes and Diabetes Prevention. Drugs 75 (10), 1071–1094. 10.1007/s40265-015-0416-8 26059289 PMC4498279

[B39] Hu C. Jia W. (2018). Diabetes in China: Epidemiology and Genetic Risk Factors and Their Clinical Utility in Personalized Medication. Diabetes 67 (1), 3–11. 10.2337/dbi17-0013 29263166

[B40] Hu S. Wang S. Fanelli B. Bell P. A. Dunning B. E. Geisse S. (2000). Pancreatic Beta-Cell K(ATP) Channel Activity and Membrane-Binding Studies with Nateglinide: A Comparison with Sulfonylureas and Repaglinide. J. Pharmacol. Exp. Ther. 293 (2), 444–452. 10773014

[B41] Hunter R. W. Hughey C. C. Lantier L. Sundelin E. I. Peggie M. Zeqiraj E. (2018). Metformin Reduces Liver Glucose Production by Inhibition of Fructose-1-6-Bisphosphatase. Nat. Med. 24 (9), 1395–1406. 10.1038/s41591-018-0159-7 30150719 PMC6207338

[B42] Husain M. Bain S. C. Jeppesen O. K. Lingvay I. Sørrig R. Treppendahl M. B. (2020). Semaglutide (SUSTAIN and PIONEER) Reduces Cardiovascular Events in Type 2 Diabetes across Varying Cardiovascular Risk. Diabetes Obes. Metab. 22 (3), 442–451. 10.1111/dom.13955 31903692 PMC7064975

[B43] Husain M. Birkenfeld A. L. Donsmark M. Dungan K. Eliaschewitz F. G. Franco D. R. PIONEER 6 Investigators (2019). Oral Semaglutide and Cardiovascular Outcomes in Patients with Type 2 Diabetes. N. Engl. J. Med. 381 (9), 841–851. 10.1056/NEJMoa1901118 31185157

[B44] International Diabetes Federation (2021). IDF Diabetes Atlas. 10th edn. Brussels, Belgium. Available at: https://www.diabetesatlas.org (Accessed December 2, 2021).

[B45] Irons B. K. Minze M. G. (2014). Drug Treatment of Type 2 Diabetes Mellitus in Patients for Whom Metformin Is Contraindicated. Diabetes Metab. Syndr. Obes. 7, 15–24. 10.2147/DMSO.S38753 24465132 PMC3900315

[B46] Ito H. Shinozaki M. Nishio S. Abe M. (2016). SGLT2 Inhibitors in the Pipeline for the Treatment of Diabetes Mellitus in Japan. Expert Opin. Pharmacother. 17 (15), 2073–2084. 10.1080/14656566.2016.1232395 27592508

[B47] Jadoon K. A. Ratcliffe S. H. Barrett D. A. Thomas E. L. Stott C. Bell J. D. (2016). Efficacy and Safety of Cannabidiol and Tetrahydrocannabivarin on Glycemic and Lipid Parameters in Patients with Type 2 Diabetes: A Randomized, Double-Blind, Placebo-Controlled, Parallel Group Pilot Study. Diabetes Care 39 (10), 1777–1786. 10.2337/dc16-0650 27573936

[B48] Jiang S. Qiu G. H. Zhu N. Hu Z. Y. Liao D. F. Qin L. (2019). ANGPTL3: a Novel Biomarker and Promising Therapeutic Target. J. Drug Target. 27 (8), 876–884. 10.1080/1061186X.2019.1566342 30615486

[B49] Jiang S. Wu X. Wang Y. Zou J. Zhao X. (2020). The Potential DPP-4 Inhibitors from Xiao-Ke-An Improve the Glucolipid Metabolism via the Activation of AKT/GSK-3β Pathway. Eur. J. Pharmacol. 882, 173272. 10.1016/j.ejphar.2020.173272 32535096

[B50] Joharapurkar A. A. Dhanesha N. A. Jain M. R. (2014). Inhibition of the Methionine Aminopeptidase 2 Enzyme for the Treatment of Obesity. Diabetes Metab. Syndr. Obes. 7, 73–84. 10.2147/DMSO.S56924 24611021 PMC3944999

[B51] Kang S. U. (2013). GPR119 Agonists: a Promising Approach for T2DM Treatment? A SWOT Analysis of GPR119. Drug Discov. Today 18 (23-24), 1309–1315. 10.1016/j.drudis.2013.09.011 24060477

[B52] Kaur P. Mittal A. Nayak S. K. Vyas M. Mishra V. Khatik G. L. (2018). Current Strategies and Drug Targets in the Management of Type 2 Diabetes Mellitus. Curr. Drug Targets 19 (15), 1738–1766. 10.2174/1389450119666180727142902 30051787

[B53] Khalse M. Bhargava A. (2018). A Review on Cardiovascular Outcome Studies of Dipeptidyl Peptidase-4 Inhibitors. Indian J. Endocrinol. Metab. 22 (5), 689–695. 10.4103/ijem.IJEM_104_18 30294582 PMC6166543

[B54] Kharroubi A. T. Darwish H. M. (2015). Diabetes Mellitus: The Epidemic of the century. World J. Diabetes 6 (6), 850–867. 10.4239/wjd.v6.i6.850 26131326 PMC4478580

[B55] Khitan Z. Kim D. H. (20132013). Fructose: a Key Factor in the Development of Metabolic Syndrome and Hypertension. J. Nutr. Metab. 2013, 682673. 10.1155/2013/682673 PMC3677638 23762544

[B56] Kim H. S. Kim D. M. Cha B. S. Park T. S. Kim K. A. Kim D. L. (2014). Efficacy of Glimepiride/metformin Fixed-Dose Combination vs Metformin Uptitration in Type 2 Diabetic Patients Inadequately Controlled on Low-Dose Metformin Monotherapy: A Randomized, Open Label, Parallel Group, Multicenter Study in Korea. J. Diabetes Investig. 5 (6), 701–708. 10.1111/jdi.12201 PMC4234234 25422771

[B57] Knowledge Sourcing Intelligence LLP (2021). Global Anti-diabetic Drugs Market (2020 to 2025) - Featuring AstraZeneca, Pfizer & Novo Nordisk Among Others. Available at: https://www.globenewswire.com/news-release/2020/05/11/2031002/0/en/Global-Anti-Diabetic-Drugs-Market-2020-to-2025-Featuring-AstraZeneca-Pfizer-Novo-Nordisk-Among-Others.html (Accessed October 5, 2021).

[B58] Kohro T. Yamazaki T. Sato H. Harada K. Ohe K. Komuro I. (2013). Trends in Antidiabetic Prescription Patterns in Japan from 2005 to 2011. Int. Heart J. 54 (2), 93–97. 10.1536/ihj.54.93 23676369

[B59] Kolodka T. Charles M. L. Raghavan A. Radichev I. A. Amatya C. Ellefson J. (2014). Preclinical Characterization of Recombinant Human Tissue Kallikrein-1 as a Novel Treatment for Type 2 Diabetes Mellitus. PLoS One 9 (8), e103981. 10.1371/journal.pone.0103981 25100328 PMC4123992

[B60] Kramer C. K. Ye C. Campbell S. Retnakaran R. (2018). Comparison of New Glucose-Lowering Drugs on Risk of Heart Failure in Type 2 Diabetes: A Network Meta-Analysis. JACC Heart Fail. 6 (10), 823–830. 10.1016/j.jchf.2018.05.021 30196071

[B61] Kulzer B. Daenschel W. Daenschel I. Schramm W. Messinger D. Weissmann J. (2018). Integrated Personalized Diabetes Management Improves Glycemic Control in Patients with Insulin-Treated Type 2 Diabetes: Results of the PDM-ProValue Study Program. Diabetes Res. Clin. Pract. 144, 200–212. 10.1016/j.diabres.2018.09.002 30205184

[B62] Lang W. Frishman W. H. (2019). Angiopoietin-Like 3 Protein Inhibition: A New Frontier in Lipid-Lowering Treatment. Cardiol. Rev. 27 (4), 211–217. 10.1097/CRD.0000000000000258 31008773

[B63] Lau J. Rousseau J. Kwon D. Bénard F. Lin K. S. (2020). A Systematic Review of Molecular Imaging Agents Targeting Bradykinin B1 and B2 Receptors. Pharmaceuticals (Basel) 13 (8). 10.3390/ph13080199 PMC7464927 32824565

[B64] Lebovitz H. E. (2011). Insulin: Potential Negative Consequences of Early Routine Use in Patients with Type 2 Diabetes. Diabetes Care 34 Suppl 2 (Suppl. 2), S225–S230. 10.2337/dc11-s225 21525460 PMC3632184

[B65] Lebovitz H. E. (2019). Thiazolidinediones: the Forgotten Diabetes Medications. Curr. Diab Rep. 19 (12), 151. 10.1007/s11892-019-1270-y 31776781 PMC6881429

[B66] Lee M. K. Kim S. G. Watkins E. Moon M. K. Rhee S. Y. Frias J. P. (2020). A Novel Non-PPARgamma Insulin Sensitizer: MLR-1023 Clinicalproof-Of-Concept in Type 2 Diabetes Mellitus. J. Diabetes Complications 34 (5), 107555. 10.1016/j.jdiacomp.2020.107555 32019723

[B67] Lee Y. S. Jun H. S. (2014). Anti-diabetic Actions of Glucagon-like Peptide-1 on Pancreatic Beta-Cells. Metabolism 63 (1), 9–19. 10.1016/j.metabol.2013.09.010 24140094

[B68] Lin Y. Sun Z. (2011). Thyroid Hormone Potentiates Insulin Signaling and Attenuates Hyperglycemia and Insulin Resistance in a Mouse Model of Type 2 Diabetes. Br. J. Pharmacol. 162 (3), 597–610. 10.1111/j.1476-5381.2010.01056.x 20883475 PMC3041250

[B69] Lipinski C. A. Reaume A. G. (2020). High Throughput *In Vivo* Phenotypic Screening for Drug Repurposing: Discovery of MLR-1023 a Novel Insulin Sensitizer and Novel Lyn Kinase Activator with Clinical Proof of Concept. Bioorg. Med. Chem. 28 (9), 115425. 10.1016/j.bmc.2020.115425 32201192

[B70] Liu R. Wang H. Xu B. Chen W. Turlova E. Dong N. (2016). Cerebrovascular Safety of Sulfonylureas: The Role of KATP Channels in Neuroprotection and the Risk of Stroke in Patients with Type 2 Diabetes. Diabetes 65 (9), 2795–2809. 10.2337/db15-1737 27207539

[B71] Lu D. Dopart R. Kendall D. A. (2016). Controlled Downregulation of the Cannabinoid CB1 Receptor Provides a Promising Approach for the Treatment of Obesity and Obesity-Derived Type 2 Diabetes. Cell Stress Chaperones 21 (1), 1–7. 10.1007/s12192-015-0653-5 26498013 PMC4679742

[B72] Lupo M. G. Ferri N. (2018). Angiopoietin-Like 3 (ANGPTL3) and Atherosclerosis: Lipid and Non-lipid Related Effects. J. Cardiovasc. Dev. Dis. 5 (3). 10.3390/jcdd5030039 PMC6162638 30011918

[B73] Lupsa B. C. Inzucchi S. E. (2018). Use of SGLT2 Inhibitors in Type 2 Diabetes: Weighing the Risks and Benefits. Diabetologia 61 (10), 2118–2125. 10.1007/s00125-018-4663-6 30132031

[B74] Marchand L. Pecquet M. Luyton C. (2020). Type 1 Diabetes Onset Triggered by COVID-19. Acta Diabetol. 57 (10), 1265–1266. 10.1007/s00592-020-01570-0 32653960 PMC7353822

[B75] Marso S. P. Bain S. C. Consoli A. Eliaschewitz F. G. Jódar E. Leiter L. A. (2016). SUSTAIN-6 InvestigatorsSemaglutide and Cardiovascular Outcomes in Patients with Type 2 Diabetes. N. Engl. J. Med. 375 (19), 1834–1844. 10.1056/NEJMoa1607141 27633186

[B76] Martens P. Mathieu C. Verbrugge F. H. (2017). Promise of SGLT2 Inhibitors in Heart Failure: Diabetes and beyond. Curr. Treat. Options. Cardiovasc. Med. 19 (3), 23. 10.1007/s11936-017-0522-x 28299616

[B77] Mathiesen D. S. Bagger J. I. Bergmann N. C. Lund A. Christensen M. B. Vilsbøll T. (2019). The Effects of Dual GLP-1/GIP Receptor Agonism on Glucagon Secretion-A Review. Int. J. Mol. Sci. 20 (17). 10.3390/ijms20174092 PMC6747202 31443356

[B78] Matschinsky F. M. (2013). GKAs for Diabetes Therapy: Why No Clinically Useful Drug after Two Decades of Trying? Trends Pharmacol. Sci. 34 (2), 90–99. 10.1016/j.tips.2012.11.007 23305809

[B79] Meier J. J. Gallwitz B. Siepmann N. Holst J. J. Deacon C. F. Schmidt W. E. (2003). Gastric Inhibitory Polypeptide (GIP) Dose-Dependently Stimulates Glucagon Secretion in Healthy Human Subjects at Euglycaemia. Diabetologia 46 (6), 798–801. 10.1007/s00125-003-1103-y 12764578

[B80] Miller B. R. Nguyen H. Hu C. J. Lin C. Nguyen Q. T. (2014). New and Emerging Drugs and Targets for Type 2 Diabetes: Reviewing the Evidence. Am. Health Drug Benefits 7 (8), 452–463. 25558307 PMC4280522

[B81] Mirabelli M. Chiefari E. Puccio L. Foti D. P. Brunetti A. (2020). Potential Benefits and Harms of Novel Antidiabetic Drugs during COVID-19 Crisis. Int. J. Environ. Res. Public Health 17 (10). 10.3390/ijerph17103664 PMC7277613 32456064

[B82] Mohan V. Khunti K. Chan S. P. Filho F. F. Tran N. Q. Ramaiya K. (2020). Management of Type 2 Diabetes in Developing Countries: Balancing Optimal Glycaemic Control and Outcomes with Affordability and Accessibility to Treatment. Diabetes Ther. 11 (1), 15–35. 10.1007/s13300-019-00733-9 PMC6965543 31773420

[B83] Mordor Intelligence (2021). Oral Anti Diabetic Drugs Market | 2021 - 26 | Industry Share, Size, Growth. Available at: https://www.mordorintelligence.com/industry-reports/oral-anti-diabetic-drug-market (Accessed October 5, 2021).

[B84] Morgen M. Jöst C. Malz M. Janowski R. Niessing D. Klein C. D. (2016). Spiroepoxytriazoles Are Fumagillin-like Irreversible Inhibitors of MetAP2 with Potent Cellular Activity. ACS Chem. Biol. 11 (4), 1001–1011. 10.1021/acschembio.5b00755 26686773

[B85] Musi N. Hirshman M. F. Nygren J. Svanfeldt M. Bavenholm P. Rooyackers O. (2002). Metformin Increases AMP-Activated Protein Kinase Activity in Skeletal Muscle of Subjects with Type 2 Diabetes. Diabetes 51 (7), 2074–2081. 10.2337/diabetes.51.7.2074 12086935

[B86] Nauck M. A. Meier J. J. (2018). Incretin Hormones: Their Role in Health and Disease. Diabetes Obes. Metab. 20 (Suppl. 1), 5–21. 10.1111/dom.13129 29364588

[B87] Ndisang J. F. Vannacci A. Rastogi S. (2017). Insulin Resistance, Type 1 and Type 2 Diabetes, and Related Complications 2017. J. Diabetes Res. 2017, 1478294. 10.1155/2017/1478294 29279853 PMC5723935

[B88] Neelamkavil S. F. Stamford A. W. Kowalski T. Biswas D. Boyle C. Chackalamannil S. (2018). Discovery of MK-8282 as a Potent G-Protein-Coupled Receptor 119 Agonist for the Treatment of Type 2 Diabetes. ACS Med. Chem. Lett. 9 (5), 457–461. 10.1021/acsmedchemlett.8b00073 29795759 PMC5949837

[B89] O'Brien M. J. Karam S. L. Wallia A. Kang R. H. Cooper A. J. Lancki N. (2018). Association of Second-Line Antidiabetic Medications with Cardiovascular Events Among Insured Adults with Type 2 Diabetes. JAMA Netw. Open 1 (8), e186125. 10.1001/jamanetworkopen.2018.6125 30646315 PMC6324353

[B90] Ochman A. R. Lipinski C. A. Handler J. A. Reaume A. G. Saporito M. S. (2012). The Lyn Kinase Activator MLR-1023 Is a Novel Insulin Receptor Potentiator that Elicits a Rapid-Onset and Durable Improvement in Glucose Homeostasis in Animal Models of Type 2 Diabetes. J. Pharmacol. Exp. Ther. 342 (1), 23–32. 10.1124/jpet.112.192187 22431203

[B91] Oki T. Matsui T. Osajima Y. (1999). Inhibitory Effect of Alpha-Glucosidase Inhibitors Varies According to its Origin. J. Agric. Food Chem. 47 (2), 550–553. 10.1021/jf980788t 10563931

[B92] Overton H. A. Fyfe M. C. Reynet C. (2008). GPR119, a Novel G Protein-Coupled Receptor Target for the Treatment of Type 2 Diabetes and Obesity. Br. J. Pharmacol. 153 Suppl 1 (Suppl. 1), S76–S81. 10.1038/sj.bjp.0707529 18037923 PMC2268073

[B93] Pappachan J. M. Fernandez C. J. Chacko E. C. (2019). Diabesity and Antidiabetic Drugs. Mol. Aspects Med. 66, 3–12. 10.1016/j.mam.2018.10.004 30391234

[B94] Petersen M. C. Shulman G. I. (2018). Mechanisms of Insulin Action and Insulin Resistance. Physiol. Rev. 98 (4), 2133–2223. 10.1152/physrev.00063.2017 30067154 PMC6170977

[B95] Pop L. M. Lingvay I. (2017). The Infamous, Famous Sulfonylureas and Cardiovascular Safety: Much Ado about Nothing? Curr. Diab Rep. 17, 124. 10.1007/s11892-017-0954-4 29063276

[B96] Quianzon C. C. Cheikh I. E. (2012). History of Current Non-insulin Medications for Diabetes Mellitus. J. Community Hosp. Intern. Med. Perspect. 2 (2). 10.3402/jchimp.v2i2.1870110.3402/jchimp.v2i3.19081 PMC3714066 23882374

[B97] Ramzan S. Timmins P. Hasan S. S. Babar Z. U. (2019). Trends in Global Prescribing of Antidiabetic Medicines in Primary Care: A Systematic Review of Literature between 2000-2018. Prim. Care Diabetes 13 (5), 409–421. 10.1016/j.pcd.2019.05.009 31213359

[B98] Rao A. D. Kuhadiya N. Reynolds K. Fonseca V. A. (2008). Is the Combination of Sulfonylureas and Metformin Associated with an Increased Risk of Cardiovascular Disease or All-Cause Mortality?: a Meta-Analysis of Observational Studies. Diabetes Care 31 (8), 1672–1678. 10.2337/dc08-0167 18458139 PMC2494623

[B99] Rask-Andersen M. Masuram S. Schiöth H. B. (2014). The Druggable Genome: Evaluation of Drug Targets in Clinical Trials Suggests Major Shifts in Molecular Class and Indication. Annu. Rev. Pharmacol. Toxicol. 54, 9–26. 10.1146/annurev-pharmtox-011613-135943 24016212

[B100] Rena G. Hardie D. G. Pearson E. R. (2017). The Mechanisms of Action of Metformin. Diabetologia 60 (9), 1577–1585. 10.1007/s00125-017-4342-z 28776086 PMC5552828

[B101] Ritter K. Buning C. Halland N. Pöverlein C. Schwink L. (2016). G Protein-Coupled Receptor 119 (GPR119) Agonists for the Treatment of Diabetes: Recent Progress and Prevailing Challenges. J. Med. Chem. 59 (8), 3579–3592. 10.1021/acs.jmedchem.5b01198 26512410

[B102] Röhrborn D. Wronkowitz N. Eckel J. (2015). DPP4 in Diabetes. Front. Immunol. 6, 386. 10.3389/fimmu.2015.00386 26284071 PMC4515598

[B103] Sano M. (2019). Mechanism by Which Dipeptidyl Peptidase-4 Inhibitors Increase the Risk of Heart Failure and Possible Differences in Heart Failure Risk. J. Cardiol. 73 (1), 28–32. 10.1016/j.jjcc.2018.07.004 30318179

[B104] Saponaro F. Sestito S. Runfola M. Rapposelli S. Chiellini G. (2020). Selective Thyroid Hormone Receptor-Beta (TRβ) Agonists: New Perspectives for the Treatment of Metabolic and Neurodegenerative Disorders. Front. Med. (Lausanne) 7 (7), 331. 10.3389/fmed.2020.00331 32733906 PMC7363807

[B105] Saporito M. S. Ochman A. R. Lipinski C. A. Handler J. A. Reaume A. G. (2012). MLR-1023 Is a Potent and Selective Allosteric Activator of Lyn Kinase *In Vitro* that Improves Glucose Tolerance *In Vivo* . J. Pharmacol. Exp. Ther. 342 (1), 15–22. 10.1124/jpet.112.192096 22473614

[B106] Scheen A. J. Paquot N. Lefèbvre P. J. (2017). Investigational Glucagon Receptor Antagonists in Phase I and II Clinical Trials for Diabetes. Expert Opin. Investig. Drugs 26 (12), 1373–1389. 10.1080/13543784.2017.1395020 29052441

[B107] Schramm T. K. Gislason G. H. Vaag A. Rasmussen J. N. Folke F. Hansen M. L. (2011). Mortality and Cardiovascular Risk Associated with Different Insulin Secretagogues Compared with Metformin in Type 2 Diabetes, with or without a Previous Myocardial Infarction: a Nationwide Study. Eur. Heart J. 32 (15), 1900–1908. 10.1093/eurheartj/ehr077 21471135

[B108] Secrest M. H. Azoulay L. Dahl M. Clemens K. K. Durand M. Hu N. (2020). A Population-Based Analysis of Antidiabetic Medications in Four Canadian Provinces: Secular Trends and Prescribing Patterns. Pharmacoepidemiol. Drug Saf. 29 (Suppl. 1), 86–92. 10.1002/pds.4878 31464069

[B109] Sharma D. Verma S. Vaidya S. Kalia K. Tiwari V. (2018). Recent Updates on GLP-1 Agonists: Current Advancements & Challenges. Biomed. Pharmacother. 108, 952–962. 10.1016/j.biopha.2018.08.088 30372907

[B110] Shukla R. Basu A. K. Mandal B. Mukhopadhyay P. Maity A. Chakraborty S. (2019). 11β Hydroxysteroid Dehydrogenase - 1 Activity in Type 2 Diabetes Mellitus: a Comparative Study. BMC Endocr. Disord. 19 (1), 15. 10.1186/s12902-019-0344-9 30678666 PMC6345010

[B111] Siddik M. A. B. Das B. C. Weiss L. Dhurandhar N. V. Hegde V. (2019). A MetAP2 Inhibitor Blocks Adipogenesis, yet Improves Glucose Uptake in Cells. Adipocyte 8 (1), 240–253. 10.1080/21623945.2019.1636627 31264515 PMC6768232

[B112] Singla R. Bindra J. Singla A. Gupta Y. Kalra S. (2019). Drug Prescription Patterns and Cost Analysis of Diabetes Therapy in India: Audit of an Endocrine Practice. Indian J. Endocrinol. Metab. 23 (1), 40–45. 10.4103/ijem.IJEM_646_18 31016151 PMC6446683

[B113] Sivitz W. I. Yorek M. A. (2010). Mitochondrial Dysfunction in Diabetes: from Molecular Mechanisms to Functional Significance and Therapeutic Opportunities. Antioxid. Redox Signal. 12 (4), 537–577. 10.1089/ars.2009.2531 19650713 PMC2824521

[B114] Sola D. Rossi L. Schianca G. P. Maffioli P. Bigliocca M. Mella R. (2015). Sulfonylureas and Their Use in Clinical Practice. Arch. Med. Sci. 11 (4), 840–848. 10.5114/aoms.2015.53304 26322096 PMC4548036

[B115] Srinivasan S. Yee S. W. Giacomini K. M. (2018). Pharmacogenetics of Antidiabetic Drugs. Adv. Pharmacol. 83, 361–389. 10.1016/bs.apha.2018.04.005 29801583 PMC10999281

[B116] Statista (2021). Major Diabetes Drugs by Sales 2020 and 2024. Available at: https://www.statista.com/statistics/1092916/major-diabetes-drugs-by-sales/ (Accessed October 5, 2021).

[B117] Steinberg G. R. Carling D. (2019). AMP-activated Protein Kinase: the Current Landscape for Drug Development. Nat. Rev. Drug Discov. 18 (7), 527–551. 10.1038/s41573-019-0019-2 30867601

[B118] Sulis P. M. Dambrós B. F. Mascarello A. Dos Santos A. R. S. Yunes R. A. Nunes R. J. (2019). Sulfonyl(thio)urea Derivative Induction of Insulin Secretion Is Mediated by Potassium, Calcium, and Sodium Channel Signal Transduction. J. Cel Physiol 234 (7), 10138–10147. 10.1002/jcp.27680 30417369

[B119] Szewczyk A. (1997). Intracellular Targets for Antidiabetic Sulfonylureas and Potassium Channel Openers. Biochem. Pharmacol. 54 (9), 961–965. 10.1016/s0006-2952(97)00136-6 9374415

[B120] Taira M. Takasu N. Komiya I. Taira T. Tanaka H. (2000). Voglibose Administration before the Evening Meal Improves Nocturnal Hypoglycemia in Insulin-dependent Diabetic Patients with Intensive Insulin Therapy. Metabolism 49 (4), 440–443. 10.1016/s0026-0495(00)80005-0 10778865

[B121] Taylor S. I. Blau J. E. Rother K. I. (2015). SGLT2 Inhibitors May Predispose to Ketoacidosis. J. Clin. Endocrinol. Metab. 100 (8), 2849–2852. 10.1210/jc.2015-1884 26086329 PMC4525004

[B122] Tian Y. A. Johnson G. Ashcroft S. J. (1998). Sulfonylureas Enhance Exocytosis from Pancreatic Beta-Cells by a Mechanism that Does Not Involve Direct Activation of Protein Kinase C. Diabetes 47 (11), 1722–1726. 10.2337/diabetes.47.11.1722 9792541

[B123] Toulis K. A. Nirantharakumar K. Pourzitaki C. Barnett A. H. Tahrani A. A. (2020). Glucokinase Activators for Type 2 Diabetes: Challenges and Future Developments. Drugs 80 (5), 467–475. 10.1007/s40265-020-01278-z 32162273

[B124] Trask L. E. Kasid N. Homa K. Chaidarun S. (2013). Safety and Efficacy of the Nonsystemic Chewable Complex Carbohydrate Dietary Supplement PAZ320 on Postprandial Glycemia when Added to Oral Agents or Insulin in Patients with Type 2 Diabetes Mellitus. Endocr. Pract. 19 (4), 627–632. 10.4158/EP12327.OR 23425645

[B125] UK Prospective Diabetes Study (UKPDS) Group (1998). Effect of Intensive Blood-Glucose Control with Metformin on Complications in Overweight Patients with Type 2 Diabetes (UKPDS 34). UK Prospective Diabetes Study (UKPDS) Group. Lancet 352 (9131), 854–865. 9742977

[B126] van den Boom L. Kaiser M. Kostev K. (2020). Prevalence of Insulin as a First-Line Therapy and Associated Factors in People with Type 2 Diabetes in German Primary Care Practices. Diabet Med. 37 (8), 1333–1339. 10.1111/dme.14338 32506471

[B127] Veiseh O. Tang B. C. Whitehead K. A. Anderson D. G. Langer R. (2015). Managing Diabetes with Nanomedicine: Challenges and Opportunities. Nat. Rev. Drug Discov. 14 (1), 45–57. 10.1038/nrd4477 25430866 PMC4751590

[B128] Wang X. Zheng P. Huang G. Yang L. Zhou Z. (2018). Dipeptidyl peptidase-4(DPP-4) Inhibitors: Promising New Agents for Autoimmune Diabetes. Clin. Exp. Med. 18 (4), 473–480. 10.1007/s10238-018-0519-0 30022375

[B129] Wang Y. An H. Liu T. Qin C. Sesaki H. Guo S. (2019). Metformin Improves Mitochondrial Respiratory Activity through Activation of AMPK. Cell Rep 29 (6), 1511–e5. 10.1016/j.celrep.2019.09.070 31693892 PMC6866677

[B130] Wentworth J. M. Colman P. G. Zafgen Study Group (2020). The Methionine Aminopeptidase 2 Inhibitor ZGN-1061 Improves Glucose Control and Weight in Overweight and Obese Individuals with Type 2 Diabetes: A Randomized, Placebo-Controlled Trial. Diabetes Obes. Metab. 22 (7), 1215–1219. 10.1111/dom.14009 32077231

[B131] Wilkinson S. Douglas I. Stirnadel-Farrant H. Fogarty D. Pokrajac A. Smeeth L. (2018). Changing Use of Antidiabetic Drugs in the UK: Trends in Prescribing 2000-2017. BMJ Open 8 (7), e022768. 10.1136/bmjopen-2018-022768 PMC6067400 30056393

[B132] Williams D. M. Nawaz A. Evans M. (2020). Drug Therapy in Obesity: A Review of Current and Emerging Treatments. Diabetes Ther. 11 (6), 1199–1216. 10.1007/s13300-020-00816-y 32297119 PMC7261312

[B133] Wu C. T. Lidsky P. V. Xiao Y. Lee I. T. Cheng R. Nakayama T. (2021). SARS-CoV-2 Infects Human Pancreatic β Cells and Elicits β Cell Impairment. Cel Metab 33 (8), 1565–e5. e5. 10.1016/j.cmet.2021.05.013 PMC8130512 34081912

[B134] Wu J. H. Foote C. Blomster J. Toyama T. Perkovic V. Sundström J. (2016). Effects of Sodium-Glucose Cotransporter-2 Inhibitors on Cardiovascular Events, Death, and Major Safety Outcomes in Adults with Type 2 Diabetes: a Systematic Review and Meta-Analysis. Lancet Diabetes Endocrinol. 4 (5), 411–419. 10.1016/S2213-8587(16)00052-8 27009625

[B135] Wu P. C. Wu V. C. Lin C. J. Pan C. F. Chen C. Y. Huang T. M. NRPB Kidney Consortium (2017). Meglitinides Increase the Risk of Hypoglycemia in Diabetic Patients with Advanced Chronic Kidney Disease: a Nationwide, Population-Based Study. Oncotarget 8 (44), 78086–78095. 10.18632/oncotarget.17475 29100450 PMC5652839

[B136] Yamamoto-Honda R. Takahashi Y. Mori Y. Yamashita S. Yoshida Y. Kawazu S. (2018). Changes in Antidiabetic Drug Prescription and Glycemic Control Trends in Elderly Patients with Type 2 Diabetes Mellitus from 2005-2013: An Analysis of the National Center Diabetes Database (NCDD-03). Intern. Med. 57 (9), 1229–1240. 10.2169/internalmedicine.9481-17 29279487 PMC5980802

[B137] Yau H. Rivera K. Lomonaco R. Cusi K. (2013). The Future of Thiazolidinedione Therapy in the Management of Type 2 Diabetes Mellitus. Curr. Diab Rep. 13 (3), 329–341. 10.1007/s11892-013-0378-8 23625197

[B138] Yoon K. H. Kang J. Kwon S. C. Trautmann M. E. Hompesch M. Stewart J. (2020). Pharmacokinetic and Dose-Finding Studies on Efpeglenatide in Patients with Type 2 Diabetes. Diabetes Obes. Metab. 22 (8), 1292–1301. 10.1111/dom.14032 32175655 PMC7383501

[B139] Yu J. G. Javorschi S. Hevener A. L. Kruszynska Y. T. Norman R. A. Sinha M. (2002). The Effect of Thiazolidinediones on Plasma Adiponectin Levels in normal, Obese, and Type 2 Diabetic Subjects. Diabetes 51 (10), 2968–2974. 10.2337/diabetes.51.10.2968 12351435

[B140] Zambad S. P. Munshi S. Dubey A. Gupta R. Busiello R. A. Lanni A. (2011). TRC150094 Attenuates Progression of Nontraditional Cardiovascular Risk Factors Associated with Obesity and Type 2 Diabetes in Obese ZSF1 Rats. Diabetes Metab. Syndr. Obes. 4, 5–16. 10.2147/DMSOTT.S15323 21448317 PMC3064414

[B141] Zhang J. (2018). The Diabetes Market in China. PharmExec. Available at: https://www.pharmexec.com/view/diabetes-market-china (Accessed December 2, 2021).

[B142] Zhang L. Chen Q. Li L. Kwong J. S. Jia P. Zhao P. (2016). Alpha-glucosidase Inhibitors and Hepatotoxicity in Type 2 Diabetes: a Systematic Review and Meta-Analysis. Sci. Rep. 6, 32649. 10.1038/srep32649 27596383 PMC5011653

[B143] Zhao W. Yang S. Chen J. Zhao J. Dong J. (2018). Forced Overexpression of FBP1 Inhibits Proliferation and Metastasis in Cholangiocarcinoma Cells via Wnt/β-Catenin Pathway. Life Sci. 210, 224–234. 10.1016/j.lfs.2018.09.009 30193944

[B144] Zhu G. Luo Y. Xu X. Zhang H. Zhu M. (2019). Anti-diabetic Compounds from the Seeds of Psoralea Corylifolia. Fitoterapia 139, 104373. 10.1016/j.fitote.2019.104373 31629053

