# Supplementary material for: Trends in Antidiabetic Drug Discovery: FDA Approved Drugs, New Drugs in Clinical Trials and Global Sales
Source: Front Pharmacol. 2022 Jan 19;12:807548. doi: 10.3389/fphar.2021.807548 (PMC8807560; doi:10.3389/fphar.2021.807548)
Supplement: Supplementary file 1 [file Table2.DOCX]

Supplementary Material

| **Supplementary Table 2.** Discontinued drugs for type 2 diabetes treatment | | |
| --- | --- | --- |
| **Insulin types** | **Approval date** | **Discontinued date** |
| INSULIN powder | 2006 | 2008 |
| INSULIN RECOMBINANT HUMAN | 1986 | 2009 |
| Iletin (1,2) | 1966 | 2009 |
| INSULIN ZINC SUSP PURIFIED BEEF | 1980 | 2009 |
| NOVOLIN L | 1983 | 1989 |
| SEMILENTE | 1980 | 1985 |
| VELOSULIN BR | 1999 | 2004 |
| MIXTARD HUMAN 70/30 | 1988 | 1994 |
| NOVOLIN 70/30 | 1986 | 1989 |
| NOVOLOG MIX 50/50 | 2008 | 2019 |
| NPH ILETIN I (BEEF-PORK) | 1966 | 2002 |
| PROTAMINE ZINC & ILETIN I (BEEF-PORK) | 1966 | 1992 |
| RYZODEG 70/30 | 2015 | 2018 |
| **Sulfonylureas** |  |  |
| TOLBUTAMIDE | 1957 | 2007 |
| CHLORPROPAMIDE | 1958 | 2011 |
| ACETOHEXAMIDE | 1964 | 1998 |
| TOLAZAMIDE | 1966 | 2002 |
| **Thiazolinediones** |  |  |
| ROSIGLITAZONE MALEATE | 2013 | 2014 |
| TROGLITAZONE | 1997 | 1999 |
| **Biguanide and thiazolinedione combination** |  |  |
| ACTOPLUS MET XR | 2009 | 2017 |
| METFORMIN HYDROCHLORIDE; ROSIGLITAZONE MALEATE | 2002 | 2017 |
| **Sulfonylurea and thiazolinedione combination** |  |  |
| AVANDARYL (glimepiride + rosiglitazone) | 2005 | 2015 |
| **Biguanide + meglitinide** |  |  |
| PRANDIMET | 2008 | 2017 |
